# DWARF TILLER1, a WUSCHEL-Related Homeobox Transcription Factor, Is Required for Tiller Growth in Rice

**DOI:** 10.1371/journal.pgen.1004154

**Published:** 2014-03-13

**Authors:** Wenfei Wang, Gang Li, Jun Zhao, Huangwei Chu, Wenhui Lin, Dabing Zhang, Zhiyong Wang, Wanqi Liang

**Affiliations:** 1 State Key Laboratory of Hybrid Rice, School of Life Sciences and Biotechnology, Shanghai Jiao Tong University, Shanghai, China; 2 Key Laboratory of Plant Molecular Physiology, Institute of Botany, Chinese Academy of Sciences, Beijing, China; 3 Graduate University of the Chinese Academy of Sciences, Beijing, China; 4 Department of Plant Biology, Carnegie Institution for Science, Stanford, California, United States of America; University of Minnesota, United States of America

## Abstract

Unlike many wild grasses, domesticated rice cultivars have uniform culm height and panicle size among tillers and the main shoot, which is an important trait for grain yield. However, the genetic basis of this trait remains unknown. Here, we report that *DWARF TILLER1* (*DWT1*) controls the developmental uniformity of the main shoot and tillers in rice (*Oryza sativa*). Most *dwt1* mutant plants develop main shoots with normal height and larger panicles, but dwarf tillers bearing smaller panicles compared with those of the wild type. In addition, *dwt1* tillers have shorter internodes with fewer and un-elongated cells compared with the wild type, indicating that DWT1 affects cell division and cell elongation. Map-based cloning revealed that *DWT1* encodes a WUSCHEL-related homeobox (WOX) transcription factor homologous to the *Arabidopsis* WOX8 and WOX9. The *DWT1* gene is highly expressed in young panicles, but undetectable in the internodes, suggesting that *DWT1* expression is spatially or temporally separated from its effect on the internode growth. Transcriptomic analysis revealed altered expression of genes involved in cell division and cell elongation, cytokinin/gibberellin homeostasis and signaling in *dwt1* shorter internodes. Moreover, the non-elongating internodes of *dwt1* are insensitive to exogenous gibberellin (GA) treatment, and some of the *slender rice1* (*slr1*) *dwt1* double mutant exhibits defective internodes similar to the *dwt1* single mutant, suggesting that the DWT1 activity in the internode elongation is directly or indirectly associated with GA signaling. This study reveals a genetic pathway synchronizing the development of tillers and the main shoot, and a new function of *WOX* genes in balancing branch growth in rice.

## Author Summary

Plant architecture is important for crop yield. In most plants, branches grow smaller than the main shoot, largely due to the ‘apical dominance’. However, in several cereal crops, including rice, wheat, and barley, the branches (tillers) have a height and size indistinguishable from the main shoot. The genetic basis of uniform tiller growth has remained elusive. We identified DWARF TILLER1, a WUSCHEL-related homeobox (WOX) transcription factor, as a positive regulator of tiller growth. Most *dwt1* mutant plants show normal main shoot but dwarf tillers and reduced panicle size. Tiller growth in *dwt1* appears to be inhibited by the main shoot, as removal of the main shoot releases the first tiller. The non-elongating internodes in *dwt1* show reduced cell number and cell size, while *DWT1* was mainly expressed in the panicles but not internodes, suggesting that *DWT1* plays a long distance regulatory role in promoting internode elongation. Genome-wide expression analysis revealed that the expression of genes related to cell division and elongation, as well as to homeostasis and signaling of cytokinin and gibberellin were affected in *dwt1* un-elongated internodes. This study reveals that a WOX transcription factor controls the growth uniformity of tillers and the main shoot in rice.

## Introduction

Rice is one of the most important crops in the world and feeds more than half of the world population. Thousands of years of domestication and breeding have selected many desirable traits in rice, including a plant architecture optimized for grain yield and quality. A mature rice plant has a main shoot and several lateral branches (tillers), with each bearing an inflorescence (panicle) at the apex. Despite their differences in bud initiation time, the main shoot and all tillers grow to a uniform height and flower at the same time [1]. This is in contrast to its wild progenitor (*Oryza rufipogon*) and most wild grasses, which have dominant main shoot growth. The uniform growth of tillers and the main shoot in many cultivated cereal crops, such as rice, wheat, barley, is an important agricultural trait because it ensures not only uniform grain size, but also synchronized maturation time and a uniform panicle layer which facilitates harvesting [2]. However, the genetic basis underlying the uniform development of tillers and the main shoot in these crops remains unknown.

The height of the rice culm is mainly determined by the length of the uppermost four or five internodes, which elongate rapidly after the initiation of reproductive growth. Internode elongation involves cell division in the intercalary meristem and subsequent cell elongation in the upper elongation zone [3]. Dwarf and semi-dwarf traits have increased rice yield by improving the harvest index and reducing lodging. Previous studies have demonstrated that gibberellin (GA) and brassinosteroid (BR) are two major hormones that promote internode elongation [4], [5]. However, none of the previously isolated rice mutants disrupt the uniform growth of the main shoot and tillers.

Members of the WOX8/9 subclade of homeobox (*WOX*) gene family have been shown to play important roles in region-specific transcription programs during many developmental processes. *STIMPY/WOX9* and *STIP-LIKE* (*STPL*, *WOX8*) are indispensable for embryonic patterning, shoot apical meristem maintenance, and cell proliferation during embryonic and post-embryonic development, mutants of WOX8/9 display reduced cell division and lethality during embryo and seedling developmental stages in *Arabidopsis* [6]–[8]. Homologs of *WOX8/9* in petunia and tomato plants were reported to play important roles in shaping inflorescence architecture by promoting the separation of lateral inflorescence meristems from the apical floral meristem [9],[10]. Here, we show that *DWT1*, a homolog of *Arabidopsis WOX8/9*, is required for the balanced growth of tillers and the main shoot in rice. The *dwt1* mutant exhibits an altered architecture with reduced height of tillers and over-growth of the main shoot panicle. Functional analysis revealed that DWT1 acts through a non-cell-autonomous mechanism to promote tiller growth downstream of *SLR1*. This finding reveals a *DWT1*-mediated genetic pathway synchronizing the development of tillers and the main shoot in rice.

## Results

### The *dwt1* mutant displays a dominant main shoot

**Figure 1:**
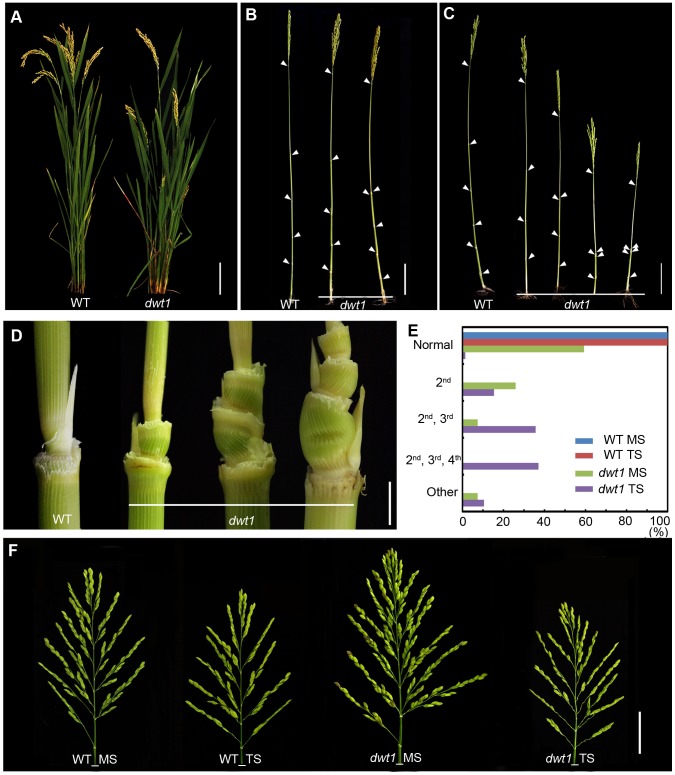
The *dwt1* mutant plants display morphological defects. A. Morphology of the wild type and *dwt1* plants after heading. Bar = 10 cm. B. Mature main shoots with leaves removed from the culm. Arrowheads point to the nodes. Bar = 10 cm. C. Mature tillers with leaves removed from the culm. Arrowheads point to the nodes. Bar = 10 cm. D. Close-up view of the internodes after heading stage. From left to right: the 2^nd^ node of the wild type, the 2^nd^ internode, the 2^nd^ and 3^rd^ internodes, the 2^nd^, 3^rd^, and 4^th^ internodes of *dwt1* mutant. Bar = 0.5 cm. E. Frequency of normal and short internodes in wild-type and *dwt1* main shoot (MS) and tiller shoot (TS). 2^nd^: only 2^nd^ internode short; 2^nd^, 3^rd^: both 2^nd^ and 3^rd^ internodes short; 2^nd^, 3^rd^, 4^th^: all 2^nd^, 3^rd^, 4^th^ internodes short. Elongation pattern of main shoots and tillers of both wild type and *dwt1* mutants were evaluated at mature stage, and 25 main shoots and 100 tillers of wild type and 25 main shoots and 143 tillers of mutant plants were observed. F. Morphology of panicles from the main shoot (MS) and tillers (TS) of wild type (WT) and *dwt1* after heading. Bar = 2 cm.

We recently identified a mutant, *dwarf tiller1* (*dwt1*), which exhibits disruption of the synchronized growth of tillers and the main shoot at maturity [11]. Genetic analysis showed that the *dwt1* mutant is a recessive allele based on the observations that the F1 progeny of backcross displays the wild-type phenotype and F2 plants segregate about 3∶1 for normal and mutant plants [11]. Unlike wild-type plants, all *dwt1* tillers form shorter culm and smaller panicles (Fig. 1A), but most *dwt1* main shoots exhibit similar height at maturity as the wild type (Fig. 1B, E). Although all the *dwt1* main shoots exhibited a normal height at maturity, 26% of the main shoots had defects in the second internode elongation and about 7% of the main shoots had shorter length of both the second and third internodes compared with the wild type (Fig. 1B, E). However a compensatory elongation of the other internodes, especially the first internode retained the same height of the *dwt1* main stem as wild-type plants (Fig. 1B, right 1).

The degree of dwarfism varied among the tillers of the same *dwt1* plant (Fig. 1 C). About 15% of *dwt1* tillers displayed a shorter length for only the second internode (22/143) (dm-type, Fig. 1C, D and E). About 36% of the tillers (51/143) had a shorter length for both the second and third internodes (Fig. 1C, D, and E), and 37% of the tillers (53/143) had a shorter length for the second, third and fourth internodes (d6-type, Fig. 1C, D and E) [12]. Even though the tiller height is dramatically reduced, the tiller number is not obviously affected in the *dwt1* mutant, and both the wild type and *dwt1* plants have about eight tillers. In addition, the short internodes appear twisted and distorted in the *dwt1* mutant at the mature developmental stage (Fig. 1D).

In contrast to the defective internode development, the *dwt1* main shoot develops a larger and denser panicle compared with that of the wild type (Fig. 1F). The average number of primary branches and secondary branches on *dwt1* main shoot panicles increases by 119% and 193%, respectively, compared to the wild-type main shoot (Table S1). Consequently, the average number of spikelets in *dwt1* main shoots increases by about 162% compared to the wild type, and the weight of 1000 grains from the *dwt1* main shoot increases by approximately 107% compared to that of the wild-type main shoot (Table S1). On the other hand, the seed weight of 1000 grains from the *dwt1* tillers decreases compared with that of wild-type tillers (Table S1). These observations suggest that *dwt1* has defects in growth uniformity between the main shoot and tillers, and its main shoot appears to be dominant compared to tillers.

**Figure 2:**
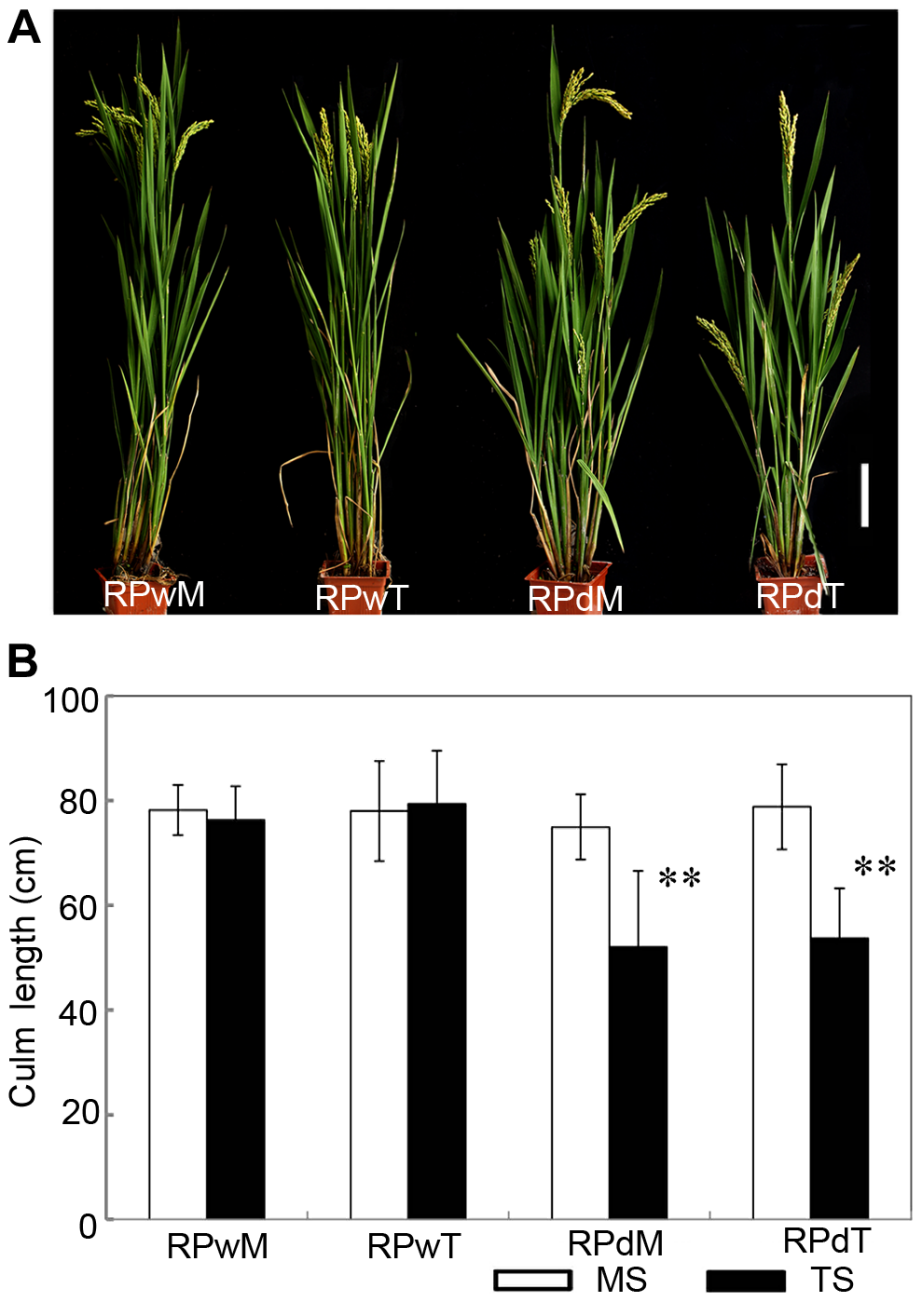
The replanted main shoot and tiller of *dwt1* reproduce the main-shoot-dominance phenotype. A. Morphology of mature plants developed from replanted main shoot of the wild type (RPwM), replanted tillers of the wild type (RPwT), replanted main shoot of *dwt1* (RPdM) and replanted tillers of *dwt1* (RPdT). Bar = 5 cm. B. The culm length of both main shoots (MS) and tillers (TS) of replanted plants. Culm length of 15 main shoots and 60 tillers of wild-type plants, 15 main shoots and 55 tillers of mutant plants were measured at mature stage. Error bars indicate SD, and the very significant differences from the wild type are marked (**p<0.01, Student's *t* test).

Suppression of lateral branches by the apex of the main shoot is a common phenomenon in plants, known as apical dominance. Elimination of apical dominance by decapitation usually promotes axillary bud outgrowth [13]. To determine whether the *dwt1* phenotype is related to apical dominance, the main shoot and each tiller of *dwt1* at the vegetative stage were separated and replanted individually in the paddy field. Each of the regenerated plants from the main shoot or tillers of *dwt1* plants developed an architecture similar to *dwt1* exhibiting dwarf tillers but a normal main shoot (Fig. 2A and B). In addition, when the main shoot of the *dwt1* plant was removed before flowering transition, the first formed tiller became a dominant shoot while subsequent tillers still remained dwarf. However, decapitation after the initiation of reproductive stage did not reestablish the main shoot dominance. Therefore we conclude that *dwt1* mutant plants have a characteristic main shoot dominance, which can be partially released by removing the developed main shoot during vegetative growth.

### *dwt1* has defects in cell proliferation and cell elongation in tiller internodes

**Figure 3:**
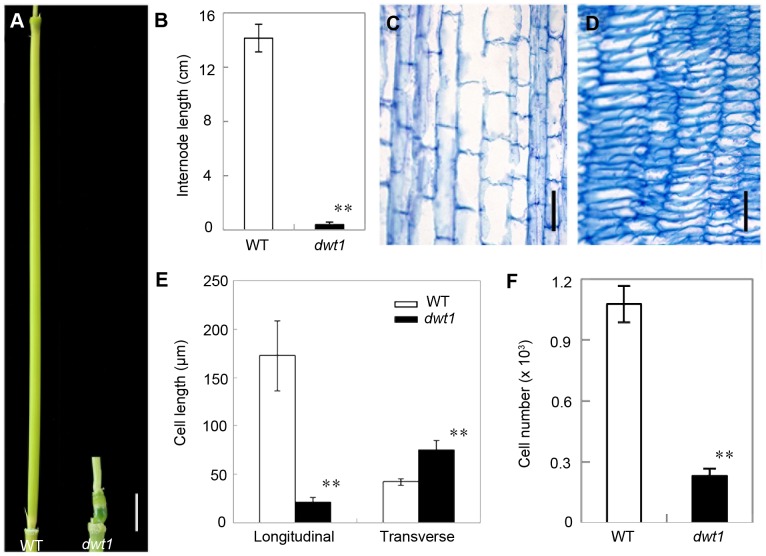
*dwt1* has defects in cell elongation and cell proliferation. A. The second internode of mature wild-type (left) and *dwt1* (right) plants. Bar = 1 cm. B. The length of second internode at mature stage. n = 30. Error bars indicate SD, and the very significant differences from wild type are marked (**p<0.01, Student's *t* test). C and D. Longitudinal sections through the middle of the second internode of the wild type (C) and *dwt1* (D) after the heading stage. Bar = 100 µm. E. Longitudinal and transverse length of the cells in the second internode. 12 second internodes from 3 individual mature plants were used, and 10 cells of each internode were measured. Error bars indicate SD, and the very significant differences from wild type are marked (**p<0.01, Student's *t* test). F. Cell number of the whole second internodes in mature plants. n = 5. Internode sample harvested from 5 individual plants were used for section. Error bars indicate SD, and the very significant differences from wild type are marked (**p<0.01, Student's *t* test).

Rice internode elongation involves cell division followed by cell elongation [3]. Because the un-elongated internodes in *dwt1* mutants are dramatically shorter (0.4±0.2 cm, n = 30) compared with wild-type plants (14.1±1.0 cm, n = 30) (Fig. 3A and B), we performed longitudinal sections of the second internodes which display obvious defective elongation in the mutant. The results show that the cells in the wild type are slender and elongated, with a cell length of about 172.6±36.2 µm (n = 30) (Fig. 3C and E). However, *dwt1* cells in the shorter internodes appear flat with a longitudinal length of about 20.9±3.7 µm (n = 30) (Fig. 3D and E). Furthermore, the *dwt1* second un-elongated internode had a dramatic reduction in cell number per internode (247±35, n = 5) in the longitudinal direction compared with the wild type (1077±90, n = 5) (Fig. 3F), suggesting that *dwt1* has defects in both cell proliferation and cell elongation along the longitudinal direction in the tiller internodes.

On the other hand, the transverse size of the un-elongated internodes in *dwt1* is abnormally larger than that of the wild type (Fig. S1). The transverse section of wild-type internodes appeared a round shape with a perimeter of about 9.8±0.2 mm (n = 10); however, the *dwt1* un-elongated internodes exhibited an elliptic shape with a perimeter of about 11.2±0.3 mm (n = 10). The increase of the width in *dwt1* results from the increase of both the cell number and cell size in radial direction. The radial cell number in un-elongated internodes of *dwt1* was about 62 cells along the major axis and 45 cells along minor axis, which is more than that in the wild type (40 cells, n = 10).

### *DWT1* encodes a WOX transcription factor

**Figure 4:**
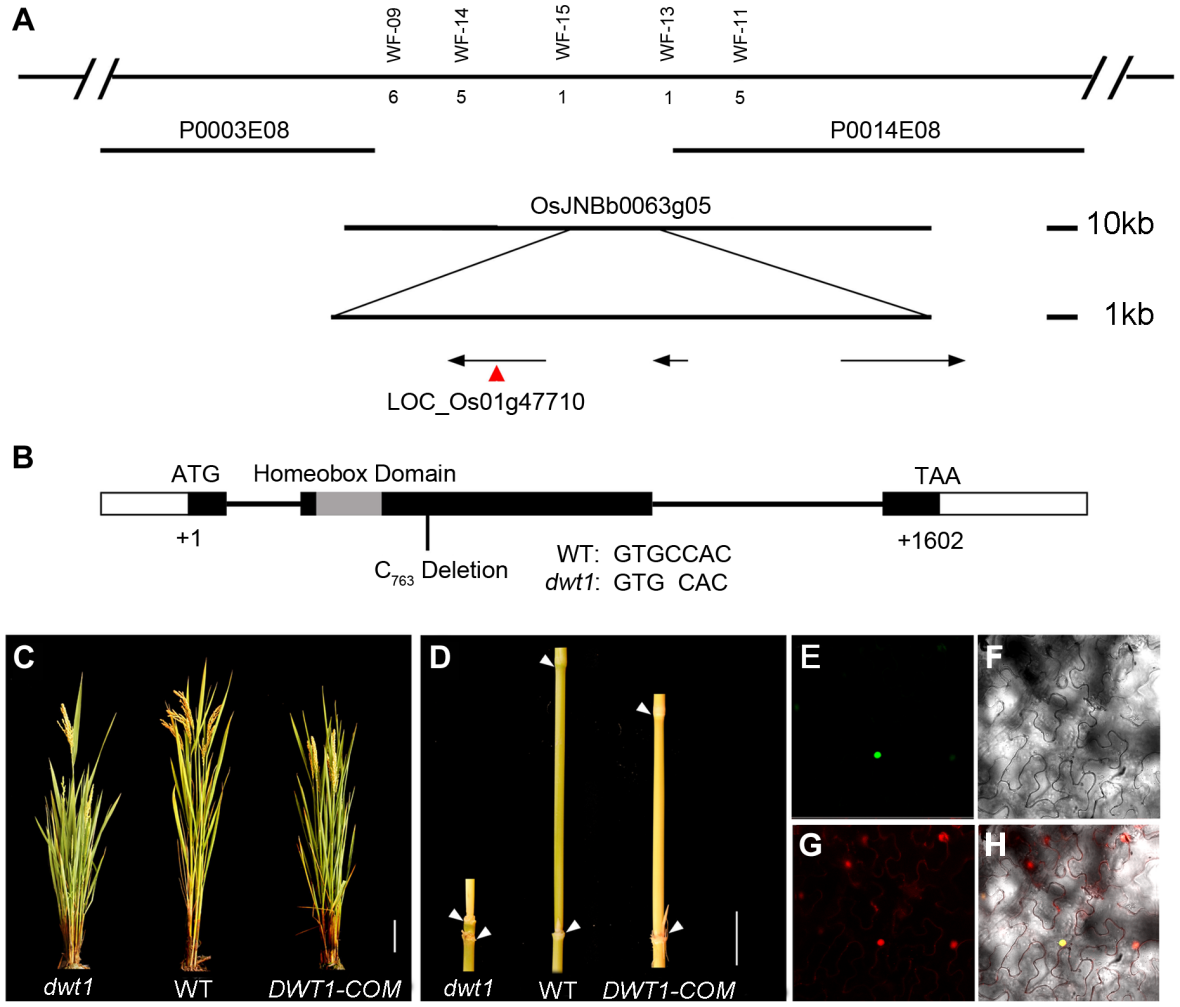
Molecular characterization of *DWT1*. A. Map-based cloning of the *DWT1* gene. The chromosomal region containing *DWT1* is diagrammed as the top line with molecular markers shown above the line. The number below the corresponding markers indicates the numbers of recombinants between the markers and *DWT1*. The BAC clones are shown as overlapping lines. B. Structure of the *DWT1* gene. The mutant sequence has one nucleotide C_763_ deletion in the second exon. Black boxes indicate exons, white boxes indicate UTRs and lines indicate introns. The grey box shows the homeobox domain. C. The plant stature of *dwt1*, the wild type, and complemented transgenic plants (DWT1-COM). Bar = 10 cm. D. The morphology of the second internodes of *dwt1*, wild type, and complemented transgenic plants (DWT1-COM). Bar = 2 cm. E–H. Nuclear localization of DWT1 protein. YFP fluorescence image (E), light view (F), PI stained image (G) and overlay of the three images (H) of *Nicotiana benthamiana* leaf epidermal cell transformed with the 35S:DWT1-YFP construct.

To identify the *DWT1* gene, we mapped the *dwt1* locus to a 22 kilo-base (kb) region on the BAC clone OSJNBb0063G05 (Fig. 4A) [11]. Three annotated open reading frames in this region were sequenced, and a single base pair deletion was observed in one of the genes, LOC_Os01g47710 (Fig. 4B), which was predicted to encode a putative *WUSCHEL*-like homeobox (WOX) protein [14]. This deletion results in a frame shift that replaces the C-terminal 279 residues with 162 new amino acids, downstream of the homeobox domain (Fig. 4B). We transformed *dwt1* plants with a 6.7-kb genomic sequence of LOC_Os01g47710. All nine transformed plants exhibited a wild-type appearance with normally elongated internodes and uniform panicle size (Fig. 4C, D), confirming that the loss-of-function mutation of this gene is responsible for the *dwt1* phenotype. Furthermore, the DWT1 protein fused with yellow fluorescent protein (YFP) is localized in the nucleus when transformed into tobacco leaves (Fig. 4E–H), consistent with the prediction of a transcription factor of WOX protein [15].

The transcribed region of the *DWT1* gene was revealed by comparing the genomic sequence with the released EST clone (OSIGCFA219G01) from the Rice *Indica* cDNA Database (RICD, http://www.ncgr.ac.cn/ricd/), and with sequences of our reverse transcription (RT)-PCR products. The predicted wild-type DWT1 protein is 533 amino acids in length and contains a conserved WUSCHEL (WUS)-like homeobox domain (amino acids 65 to 132 Fig. S2), which shares the highest sequence similarity with WOX8 and WOX9 proteins in *Arabidopsis* [14]. In addition to the homeobox domain, DWT1 also shares conserved N-terminal and C-terminal domains with WOX8/9-related proteins, but not with WUS (Fig. S2). Previous studies showed that the C-terminal domain might contribute to the dimerization of WUS proteins in *Arabidopsis* and snapdragon [16], [17]. The C-terminal deletion of DWT1 protein might disturb the dimerization of the DWT1 protein.

Phylogenetic analysis showed that the WOX8/9 subfamily of dicots and monocots are divided into two clades, and this subfamily is believed to be generated by gene duplication after the evolutionary separation of dicots and monocots (Fig. S3) [18]. In dicot plants, WOX8 and WOX9 proteins apparently arose by gene duplication from an ancestral gene. The EVERGREEN (EVG) and SISTER OF EVERGREEN (SOE) from petunia [9], and COMPOUND INFLORESCENCE in tomato [10] are more similar to STIP than STPL of *Arabidopsis*. In rice, there are three predicted WOX9 homologs, DWT1, DWT-LIKE 1 (DWL1, LOC_Os07g34880) and DWT-LIKE 2 (DWL2, LOC_Os05g48990). DWT1 and DWL1 are separated from DWL2 in two subclades with homologs of maize. These gene duplications suggest that after the divergence of monocots and eudicots, these subgroups in the WOX8/9 clade have been expanded, possibly with functional diversification (Fig. S3).

To understand the evolutionary role of *DWT1*, we did sequence analysis of *DWT1* in cultivated rice and wild rice strains. Investigation of the SNP dataset of 508 *indica* and 341 *temperate japonica* rice cultivars [19] revealed 14 SNPs in the non-coding sequence and no SNP in the coding sequence of a 4.3-Kb *DWT1*genomic region. By contrast, we observed 4 nucleotide variations between these cultivated rice and 11 wild rice strains in the coding region. A 2-bp nucleotide change in exon 2, CA (1284–1285) in cultivated rice changed to GC in 11 wild rice strains (6 *Oryza. rufipogon* and 5 *Oryza. nivara* strains), causing the amino acid change from glutamine to alanine (Fig. S4). Whether this sequence variation causes rice morphological difference between cultivated rice and wild rice remains to be elucidated.

### *DWT1* functions in a non-cell autonomous manner

**Figure 5:**
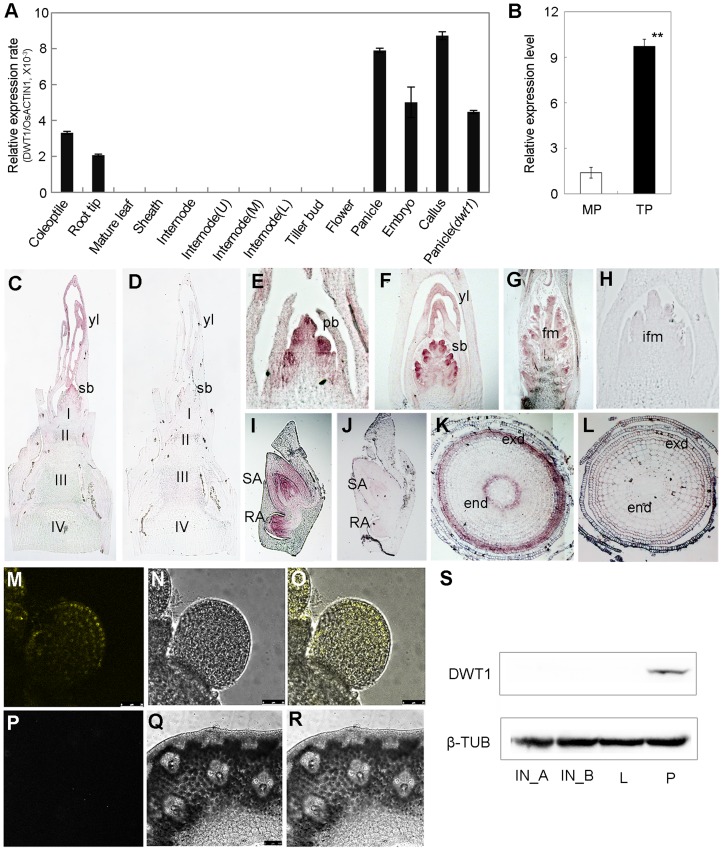
Expression pattern of DWT1 transcripts and proteins. A. qRT-PCR analysis of *DWT1* gene expression levels in the wild-type tissues including coleoptiles (36 h after seed germination), root tips, mature leaf and sheath, the second internode during elongating (1 cm in length), segments of the upper (U), middle (M), and lower (L) parts of the second internode with 3 cm in length, dormant tiller bud, panicle (less than 1 cm in length), spikelet at stage Sp8 during development of pistil [63], young embryo (10 days after fertilization), and callus with 20–days regeneration. Rice *ACTIN1* (*OsACTIN1*) was used as a control. Error bars indicate SD. n = 3. B. qRT-PCR analysis of *DWT1* gene expression levels in young panicle of wild type. This experiment was biologically repeated three times, and 30 young panicles of the main shoot (MP, Length = 5 mm), and the tiller (TP, Length = 5 mm) respectively were used in each test. Error bars indicate SD, and the significant differences from panicle of main shoot are marked (**p<0.01, Student's *t* test). C–L. *In situ* hybridization of *DWT1*. Signals were detected in the primary branch meristem (E), secondary branch meristem (C, F), top portion of the panicle (G), shoot apical and radical apical of young embryo (I), and endodermis and exodermis of root tip (K). D, H, J and L were corresponding sections hybridized with the sense probe.I,II,III,IV, the first, second, third and the forth internode, respectively; pb, primary branch meristem; sb, secondary branch meristem; yl, young leave; fm, floral meristem; ifm, inflorescence meristem; exd, exodermis layer; end, endodermis layer. M–R. YFP fluorescence image of the branch meristem (M–O) and the internode (P–R) in transgenic plants expressing *pDWT1:DWT1-YFP*. M and P are fluorescence image, N and Q are light view, O and R are overlapping of fluorescence image and light view. S. Western-blot analysis of DWT1 protein levels. DWT1 protein was analyzed by immunoblotting using an anti-DWT1 antibody using mature flag leaf (L, 3 leaves from 3 different plants), elongating internodes (IN_A, length = 0.5 cm; IN_B, length = 3 cm, 10 internodes from different plants) and young panicle (P, length less than 2 mm, 50 panicles from different plants). β-tubulin was used as a control. These experiments were biologically repeated three times.

Quantitative reverse transcription (qRT)-PCR analysis revealed the expression of *DWT1* mRNA in the callus, young panicle, young embryo, root tip and coleoptile, but not in the mature leaves, and mature spikelets (Fig. 5A). A higher expression level of *DWT1* was observed in panicles of tillers than that of the main shoot (Fig. 5B). Notably, no obvious expression signal of *DWT1* was detectable in the wild-type internodes where the *dwt1* mutation causes the most severe phenotype (Fig. 5A). Furthermore, *in situ* hybridization confirmed no detectable expression of *DWT1* in the elongating internode (Fig. 5C), but high *DWT1* expression in the panicle meristem including the primordia of primary and secondary branches, floral meristem, and leaf primordia surrounding these meristems (Fig. 5E–G). Consistent with qRT-PCR results, *DWT1* transcripts were observed in the young embryo 10 days after fertilization (Fig. 5I), and in the endodermis and exodermis layers of the elongation zone in root tip (Fig. 5K).

To further understand the function of the rice *WOX8/9* clade, we analyzed the tissue-specific expression pattern of two *DWT1* homologs. According to rice microarray data (http://bar.utoronto.ca/efprice/cgi-bin/efpWeb.cgi), *DWL2* has high expression level in the inflorescence meristem and embryo, but not in the internode. qRT-PCR analysis confirmed the expression of *DWL1* and *DWL2* in the panicle meristem but not in the internode, and no significant expression change in the *dwt1* mutant (Fig. S5). The overlapping expression pattern of *DWT1* and its two homologs suggest that the three rice *WOX8/9* homologs may have similar function as *DWT1*.

Both qRT-PCR and in situ hybridization did not detect the transcripts of *DWT1* in the internode, strongly suggesting a non-cell-autonomous manner of DWT1 function. One possibility is that DWT1 acts as a mobile protein signal, moving from the panicle meristem to the underneath internode. Alternatively, DWT1 may promote the production of a mobile signal in the panicle meristem. To test these possibilities, we detected the DWT1 protein localization in the transgenic plants harboring DWT1 protein fused with YFP (yellow fluorescence protein), driven by the native *DWT1* promoter. We could only observe the DWT1 protein in the young panicle but not in elongating internodes (Fig. 5M–R). Consistently, western-blot only detected DWT1 proteins in panicle meristem tissues (Fig. 5S), suggesting that DWT1 may regulate the internode elongation by producing a mobile signal in the panicle.

### Un-elongated internodes in *dwt1* have changed expression of genes involved in cell division and cell elongation

**Figure 6:**
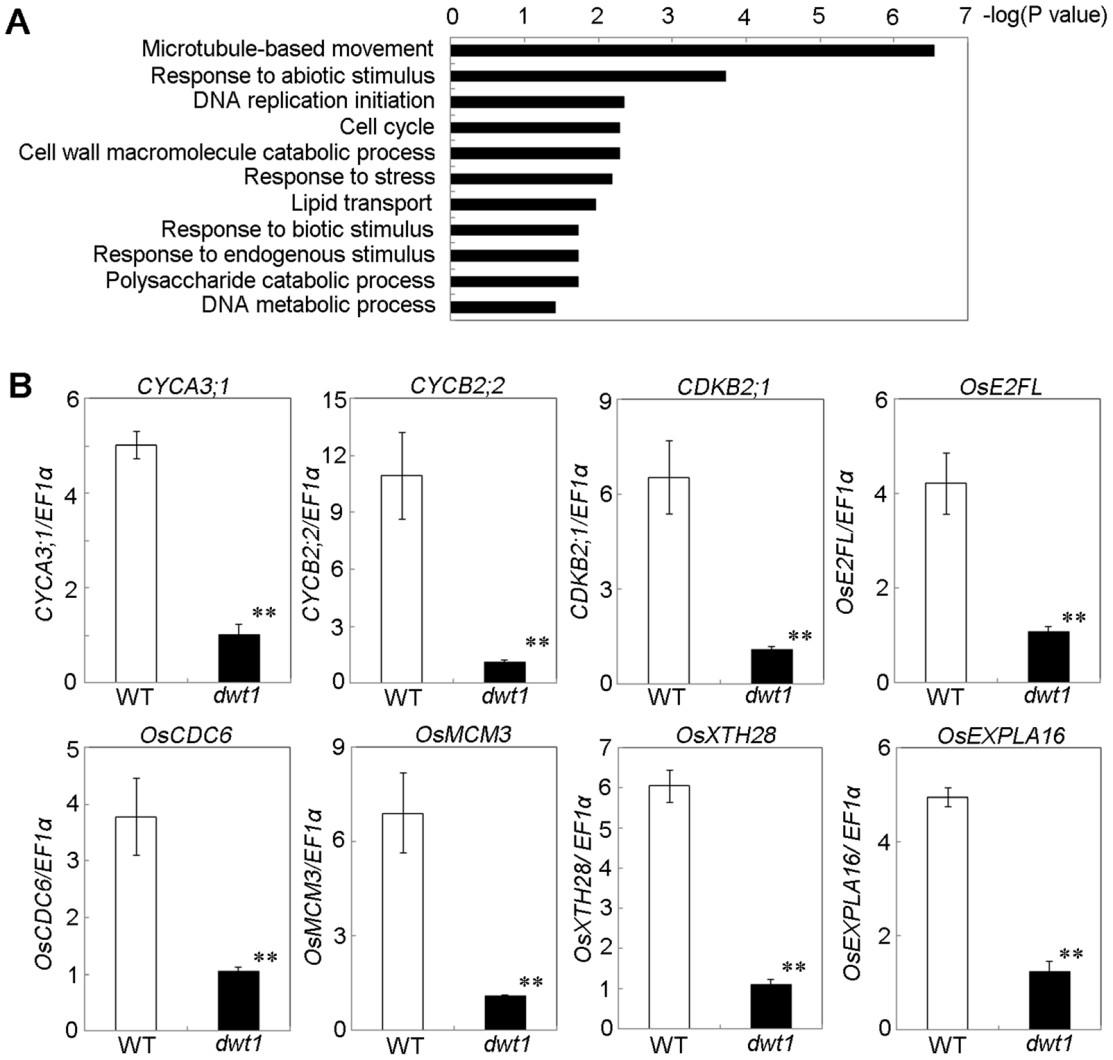
*DWT1* affects the expression of genes related to cell division and cell elongation. A. Identification of GO biological process categories for the genes differentially expressed in *dwt1* shorter internodes. The negative logarithm (base 10) of the adjusted P value was used as the bar length. B. qRT-PCR confirmed the differential expression of genes involved in cell division and cell elongation in the elongating second internode of the wild type and *dwt1*. . Rice *ELONGATION FACTOR 1 ALPHA* (*EF1α*) gene was used as a control. This experiment was biologically repeated three times, and 10 elongating second internodes of tillers (Length = 0.5 cm) were used for each biological repeat. Error bars indicate SD, and the significant differences from the wild type are marked (**p<0.01, Student's *t* test).

To understand the molecular mechanism of DWT1 in regulating rice internode development, we analyzed gene expression changes of the un-elongated second internode of *dwt1* tillers during the elongation stage using the Affymetrix rice genomic arrays. Compared with the wild type, a total of 476 genes were up-regulated and 499 genes down-regulated in *dwt1* after statistical analysis (>threefold change; P<0.001; Table S2). Functional analysis by gene ontology (GO) enrichment showed that eleven GO categories were enriched from the differential expressed genes (Fig. 6A, Table S3). The most enriched one was “microtubule-based movement”, which contains genes encoding kinesins, tubulins and other cytoskeleton related proteins. Kinesins belong to a class of microtubule-associated proteins with a motor domain for binding and moving along the microtubules. Some of these kinesin members, including *AtKINESIN5* (*ATK5*), *PHRAGMOPLAST ORIENTING KINESIN 1* and *2* (*POK1, POK2*), *KCA1* and *KCA2* are implicated in multiple mitosis-related processes, such as spindle formation, phragmoplast formation and cell plate formation [20]–[22]. Moreover, most of tubulin proteins differentially expressed are beta-tubulins, among which *OsTUB6* was previously reported as a component of the spindle skeleton in mitosis [23].

In addition, the enriched GO categories “DNA replication initiation”, “DNA metabolic process” and “cell cycle” from *dwt1* microarray data are directly related to cell division (Fig. 6A). Furthermore, reduced expression of *OsCYCA3;1, CYCLINB2;2* (*CYCB2;Os2*), *CYCLIN-DEPENDENT KINASE B2;1* (*CDKB2;1/CDC2OS3*), homologs of *CELL DIVISION CONTROL 6* (*CDC6*), *MINI CHROMOSOME MAINTENANCE* (*MCM3*) and *E2 PROMOTER-BINDING FACTOR* (*E2F*), *OsEXPA16* and *OsXTH28* were confirmed by qRT-PCR in *dwt1* (Fig. 6 B), which closely correlates with the microarray data (Pearson correlation = 0.96) (Table S2, S4). *CDC6* and *MCM3* are involved in the DNA replication under the control of the *E2F* transcription factor in *Arabidopsis* [24], [25]. *OsCYCA3;1* encodes a type-A cyclin, a homolog of tobacco *Nicta;CYCA3;2*, *CYCB2;Os2* encodes a type-B cyclin, and *CDKB2;1/CDC2OS3* encodes a cyclin-dependent kinase, which are implicated in cell cycle control [26], [27]. *OsEXPA16* encodes a α-expansin protein known to increase cell wall extensibility [28], and *OsXTH28* encodes a homolog of *XTH28*, a xyloglucan endotransglycosylase involved in cell growth in stamen filament development in *Arabidopsis* [29].

In plants, cytokinin plays a central role in promoting cell division. Cytokinin response is positively regulated by the cytokinin-inducible type-B *Response Regulators* (*RRs*) but negatively regulated by the type-A RRs [30]. Three type-A *RRs*: *OsRR6*, *OsRR9, OsRR10*, were upregulated in the shortened internode of *dwt1* (Table S5, Fig. S6). In addition, the expression of *OsCYTOKININ OXIDASE 4* (*OsCKX4*) and *OsCKX9* encoding cytokinin-inactivating enzymes was elevated in *dwt1* (Fig. S6). These results suggest that altered cytokinin signaling and reduced amount of active cytokinin may contribute to the reduced activity of cell division in *dwt1*.

### *DWT1* provides an activity required for GA promotion of cell elongation

**Figure 7:**
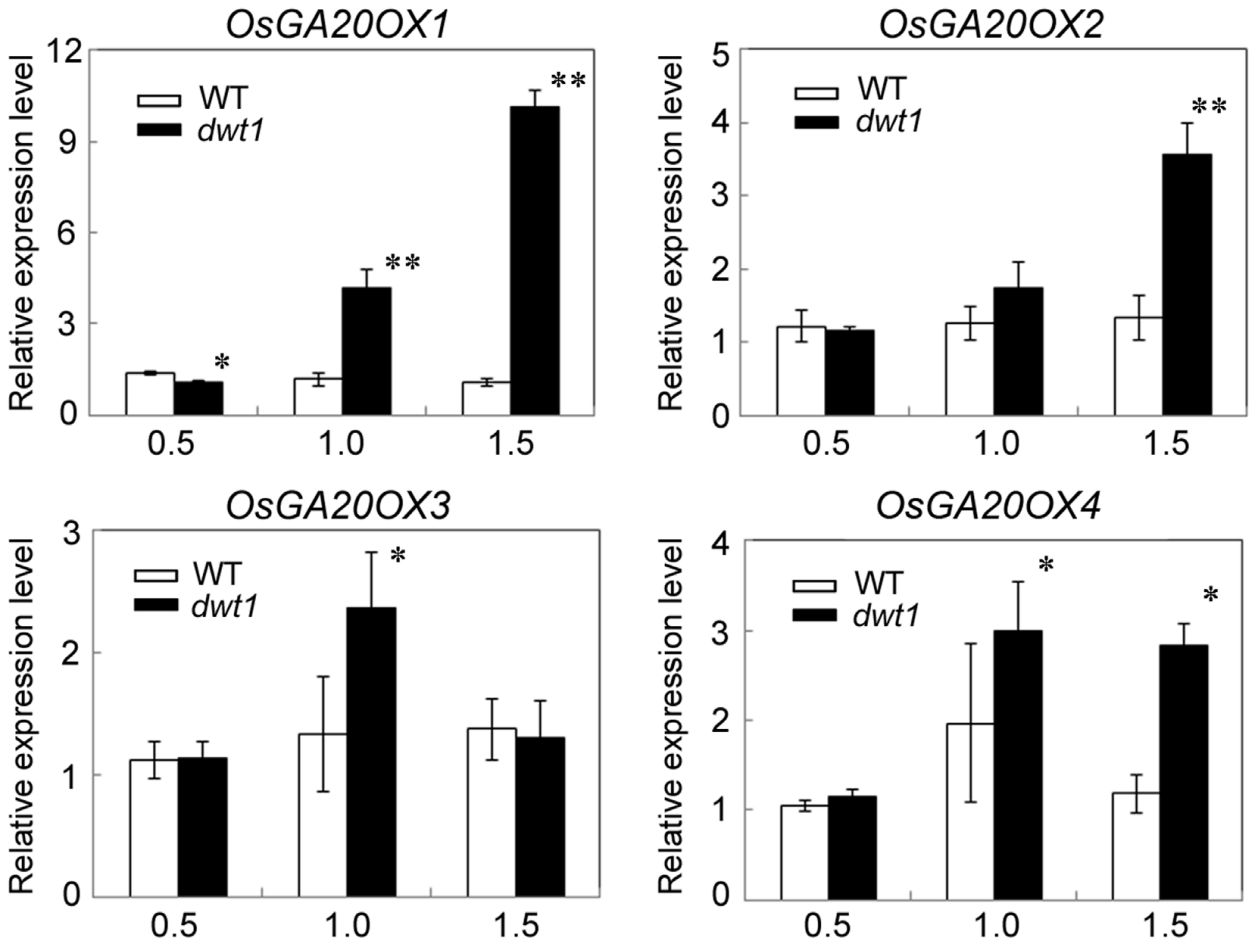
*OsGA20OX* genes have increased expression in *dwt1*. Transcript levels of *OsGA20OX1, OsGA20OX2, OsGA20OX3* and *OsGA20OX4* in the second internodes of the wild type and *dwt1*. The *EF1α* gene was used as a control. Three sequential developmental stages of elongating internode of wild type (Length = 0.5 cm, 1 cm and 3 cm) and *dwt1* at the corresponding developmental stage of wild type were analyzed. This experiment was biologically repeated three times, and 10 internodes were used for each biological repeat. Error bars indicate SD. Significant differences from the wild type are marked (*P<0.05, ** P<0.01, Student's *t* test).

Gibberellin (GA) is a crucial phytohormone in promoting the stem elongation in rice [31], [32]. We observed that the expression level of *OsENT-COPALYL DIPHOSPHATE SYNTHETASE 1* (*OsCPS1*) encoding a GA biosynthetic enzyme was reduced, and that of *OsGIBBERELLIN 2-OXIDASE 1*(*OsGA2OX1*) encoding a GA-deactivating enzyme was upregulated in the un-elongated internode of *dwt1* (Table S5). In addition, four genes encoding GA20-oxidases (*OsGA20OX1*, *OsGA20OX2*, *OsGA20OX3*, and *OsGA20OX4*), which are feedback inhibited by GA signaling, displayed increased expression in *dwt1* (Fig. 7). Therefore the developmental defects of *dwt1* internodes may be associated with altered GA homeostasis or signaling

**Figure 8:**
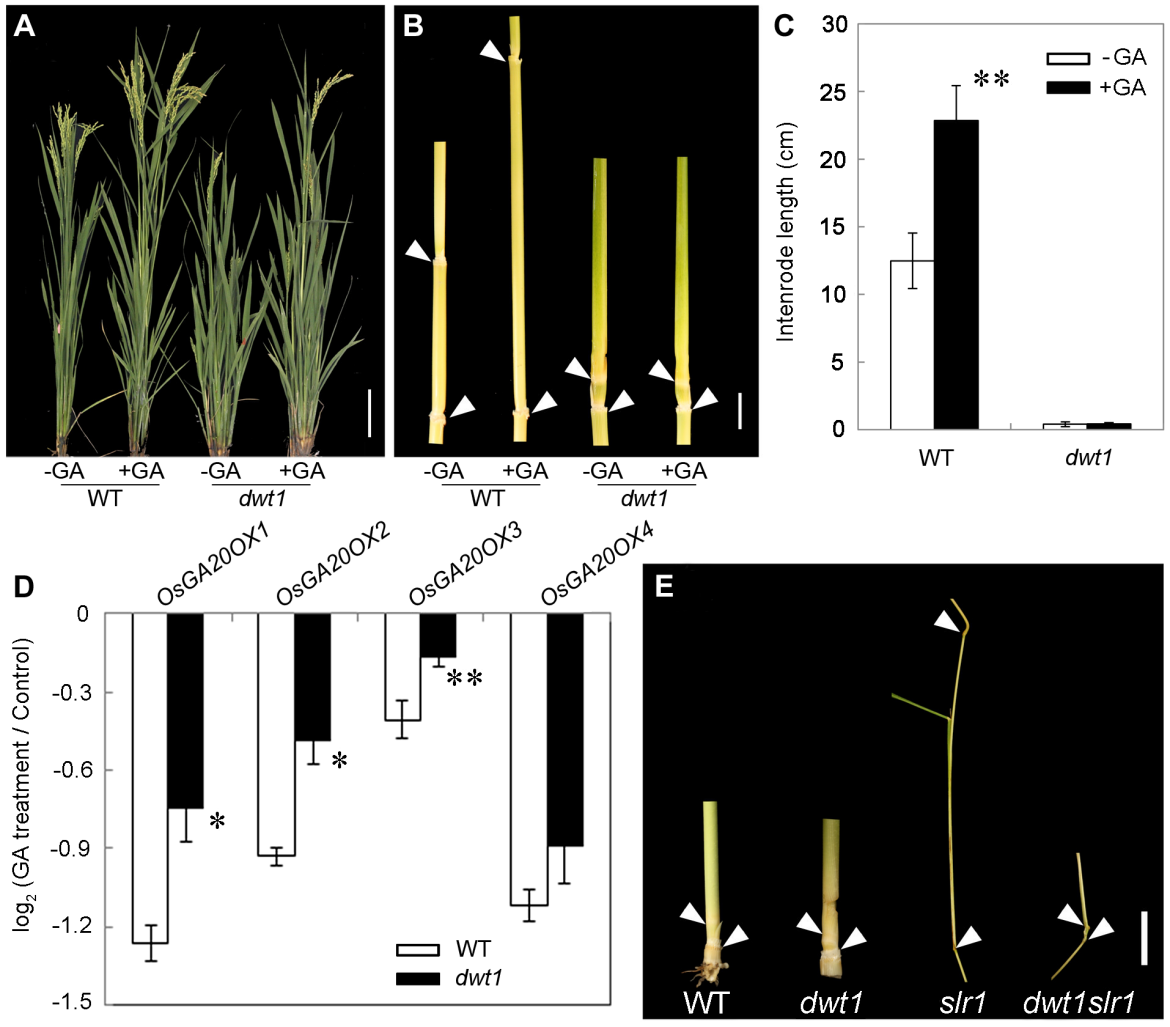
The *dwt1* tiller internodes are insensitive to GA treatment, and *DWT1* may act downstream of SLR1 in the tiller internode elongation. A. Morphological comparison of the wild type and *dwt1* treated with mock solution or 100 µM GA_3_. 20 plants were uses for each treatment and representative images are shown. Bar = 10 cm. B. The second internodes of the wild type and *dwt1* treated with mock solution or 100 µM GA_3_. Representative images of tiller internodes are shown. Bar = 1 cm. C. The internode length of the wild type and *dwt1* treated with mock solution or 100 µM GA_3_. Length of 20 tiller internodes were measured 20 days after treatment. Error bars indicate SD, and the significant differences from no GA treated control are marked (** P<0.01, Student's *t* test). D. qRT–PCR analysis of *OsGA20OX* genes in the elongating internode of the wild type and *dwt1* treated with mock solution or 100 µM GA_3_. *EF1α* gene was used as a control. This experiment was biologically repeated three times, and 5 internodes (Length = 5 mm) were used for each biological repeat. Error bars indicate SD. Significant differences from the wild type are marked (* P<0.05, ** P<0.01, Student's *t* test). E. Phenotype of basal internodes in the wild type, *dwt1*, *slr1* and *dwt1 slr1*double mutant. Arrows point to the nodes on the culm. Bar = 1 cm.

To further test whether *dwt1* has a defect in GA synthesis or signaling, we treated the wild-type and *dwt1* mutant plants with active GA_3_ after the transition to the reproductive stage. Unlike the normal response to GA of the wild-type internodes and the normal internodes in *dwt1*, the defective internodes of *dwt1* showed little response to GA (Fig. 8A, B and C, Fig. S7). Consistent with the reduced morphological response, the expression levels of *OsGA20OX1*, *OsGA20OX2*, *OsGA20OX3*, and *OsGA20OX4* were less responsive to GA treatment in *dwt1* un-elongated internodes than normal ones of the wild type (Fig. 8D), suggesting a defect in GA signaling in these internodes.

*SLENDER RICE1* (*SLR1*) is a nuclear-localized DELLA-domain protein that functions as a central suppressor of GA signaling in rice [33], [34]. Compared with the wild type, *slr1* mutants display a quick elongation of the basal internode at the seedling stage because of the constitutively activated GA response. To determine the genetic relationship between *SLR1* and *DWT1*, we crossed *dwt1* with *slr1*, and identified the double mutant by genotyping (see methods). The *slr1 dwt1* double mutant showed a subset of twisted and shorter internodes similar to *dwt1* plants (Fig. 8E, Fig. S8 C, F), while other internodes elongated as those of *slr1* mutant (Fig. S8 A, B, D, E), suggesting that the DWT1-dependent activity is required for the internode elongation in the absence of SLR1.

## Discussion

Shoot branching is one of the most important developmental processes that determine crop yields. While most wild plants have dominant main shoot and weaker branches, two extreme branching traits have been selected during the domestication of cereal crops. Some crops, such as maize and sorghum, exhibit enhanced apical dominance and suppression of branches compared to their highly branched ancestors [35]. Suppression of branch development reduces the competition for resources and thus enhances the productivity of the main shoot. In contrast, other cultivated species, including rice, wheat and barley, have been selected for multiple tillers that develop from buds at the basal un-elongated nodes of the main shoot but bearing panicles that reach similar size as the main shoot [35]. The intra-plant panicle uniformity is essential for the high yield in these species, but the underlying mechanism has remained elusive. Our study identified *DWT1*, a *WOX* member, as an essential genetic component specifying this trait in rice.

### *DWT1* represents a key switch determining rice architecture

The *WOX* genes form a plant specific clade of the homeobox transcription factor superfamily. Studies in dicot plants indicate that the *WOX8/9* subclade genes play important roles in a wide range of developmental processes, such as embryonic patterning, stem-cell maintenance, inflorescence architecture development and organ formation [8], [9], [10], [15], [36]. Rice has three *WOX* members within the *WOX8/9* subclade, and these members were assumed to be generated by duplication after the divergence of monocots from dicots [14]. Based on the functional analysis of *DWT1* in this study, we hypothesize that *DWT1* plays a key role in controlling the developmental uniformity of main shoot and tillers and may have been selected during rice domestication.

The activated tiller buds develop their own adventitious roots, and thus gain a certain degree of independence from the main shoot. It is not clear whether the tiller development is also under the control of the main shoot after this transition. The *dwt1* mutant shows not only dwarfed tillers bearing smaller panicles, but also an enhanced growth of the panicle on the main shoot, suggesting an enhanced apical dominance. It is also possible that a defect in tiller growth reduces the competition against the main shoot, resulting in quicker main shoot growth compared to wild type. The individual *dwt1* tiller separated from the main shoot is able to grow into a whole rice plant with a near normal main shoot and dwarf tillers, suggesting that *DWT1* suppresses the apical dominance in rice. Alternatively, the higher expression level of *DWT1* in tiller panicles than the main shoot panicle may provide an enhanced growth vigor to the tillers, or a higher level of growth-promoting signal to the tiller internodes, which is essential for successful competition against the main shoot.

It has been demonstrated that *TEOSINTE BRANCHED 1* (*TB1*) is the major contributor to the enhanced apical dominance during the domestication of maize. One *tb1* allele with a transposon inserted in the regulatory region was selected from the maize wild ancestor teosinte. The inserted retro-element causes a two-fold increase in *TB1* expression, which is strong enough for the transformation of a highly branched architecture of teosinte to the modern maize architecture [37]. It is not clear whether *DWT1* is a key target being selected during the domestication of rice. Sequence analysis revealed an amino acid variation in the C-terminus of DWT1 coding region between cultivated rice and wild rice strains (Fig. S4). The C-terminal domain may be required for the homo- or heterodimerization of the DWT1 protein. The effect of this sequence variation on DWT1 function remains to be elucidated.

### DWT1 enhances tiller growth vigor but does not affect tiller bud initiation

Lateral branch development involves bud formation, bud outgrowth and branch growth. In most plants, the early events of lateral bud formation and outgrowth are inhibited by the apex of the main shoot, due to the apical dominance. How growth vigor of the branch is influenced by the main shoot is less understood. Apical dominance is mediated by the biosynthesis and transport of the phytohormone auxin [38]. Auxin is transported basipetally from the apical bud and inhibits the outgrowth of lateral buds. It is proposed that the repressive effect of auxin is mediated by secondary messengers. Strigolactone is synthesized in both the roots and the shoots and transported acropetally, and it plays an important role in repressing bud growth in several species, such as pea, petunia, *Arabidopsis* and rice, possibly by reducing auxin transport in the main shoot [39]. Cytokinin, mostly synthesized in the root and transported acropetally, is able to initiate the outgrowth of lateral buds [40]–[43]. Several *WOX* genes, including the *DWT1* homologs in *Arabidopsis*, have been shown to be able to directly regulate genes involved in auxin and cytokinin biosynthesis, homeostasis, transportation and signaling. But none of them have been reported to be required for the main shoot dominance.

The *dwt1* mutant does not show an obvious difference in tiller number compared with the wild type, indicating that the initiation and outgrowth of tiller buds are not affected. Accordingly, no significant expression change of genes related to strigolactone biosynthesis and signaling pathway was observed in the leaf, root and basal shoot of *dwt1* (Fig. S9). In addition, the expression levels of cytokinin-related genes, *OsRR6*, *OsRR9, OsRR10*, *OsCKX4* and *OsCKX9*, are altered in the internodes, but not in the root or basal shoot of *dwt1* (Fig. S10), suggesting that *dwt1* does not have a defect in cytokinin signaling during lateral branch initiation and outgrowth. Thus, we propose that *DWT1* enhances tiller growth vigor at late stages after the outgrowth of tiller buds.

The higher expression level of *DWT1* and more severe phenotypes in tillers than in main stem suggest that the differential DWT1 activity counter balance the weaker growth vigor of the tillers. The main shoot enters reproductive growth a bit earlier than tillers, thus may gain growth priority over tillers. The observation that removal of main shoot releases suppression of the next tiller in *dwt1* suggests that DWT1 provides a competitive advantage to the younger tillers. The low percentage of un-elongated internodes observed in *dwt1* main shoot suggests a minor but also positive function of DWT1 in promoting internode elongation in the main shoot, consistent with the lower level of DWT1 expression in main shoot panicles compared to tillers. It is likely that DWT1 is required for promoting internode growth and the higher level of DWT1 expression in tillers enhances the growth vigor to overcome the main shoot dominance. In addition, there may be an as yet unknown main shoot factor specifically responsible for establishing the main shoot dominance independent of *DWT1* function, as a compensatory elongation of other internodes was observed in main shoots with an un-elongated internode.

### DWT1 may regulate internode growth through a mobile signal

The phenotype of reduced internodes of *dwt1* tillers seems similar to the dwarfism of mutants with defective synthesis or signaling of GA or brassinosteroids (BRs) [44]–[48]. However, no obvious expression change of genes relative to BR biosynthesis, metabolism and signaling pathways was seen in *dwt1* (Table. S4). In addition, BR related mutants display dark green, rugose, and erect leaves [45]–[47], which are not observed in *dwt1*. In addition, none of the previously reported BR-related mutants have distorted internodes or a distinction between main shoot and tillers, suggesting that the elongation defect of *dwt1* internodes is unlikely due to BR deficiency. On the other hand, the un-elongated internodes and expression of *GA20OX* genes showed insensitivity to GA treatment in *dwt1*, suggesting a defect in GA response. The *slr1* mutation was unable to suppress the dwarf-tiller phenotype of *dwt1*, indicating that the action of DWT1 on internode elongation, possibly mediated by a mobile signal, is downstream, or independent of SLR1.

The dwarfed tillers of *dwt1* show reduced cell division and cell elongation. In the shorter internode of *dwt1* mutant, not surprisingly, a set of cell cycle related genes were observed to have decreased expression, while the expression of the negative regulators of cytokinin signaling and some cytokinin-inactivating enzymes were increased. Both type-A *RR* genes and *CKX* genes have been shown to function as negative regulators in cytokinin signaling and cell division [49]. *OsWOX11* directly represses one of the type-A RR genes, *RR2*, leading to enhanced cytokinin signaling and crown root development in rice [50]. WUS directly represses the transcription of several *ARABIDOPSIS RESPONSE REGULATOR* (*ARR*) genes to positively regulate stem cells in *Arabidopsis* [51]. As no *DWT1* expression was detectable in the elongating internode, it is quite possible that the *DWT1* may indirectly repress the expression of type-A RRs by promoting a mobile signal from the apical region where *DWT1* is expressed. Alternatively, given the repressing effect of GA on the expression of type-A *ARR* genes and activation of type-A gene *ARR5* by GA response inhibitor SPINDLY (SPY) in *Arabidopsis* [52], the change of cytokinin pathway in *dwt1* mutant may result from the secondary effect of abnormal GA signaling.

*DWT1* is expressed in the panicle meristem at stages when the 2nd, 3rd and 4th internodes start to elongate [53]. Consistently, only the elongation of internodes at these positions is affected in the *dwt1* mutant, suggesting that *DWT1* has a spatiotemporal specific function. The frequency of un-elongated internodes inversely correlated with the distance from the apical meristem. The loss of the function of the apically expressed *DWT1* leads to elongation defects in 92% of the second internodes, 75% of the third internodes and only 39% of the fourth internodes. It is clear that internodes closest to the apical meristem were most affected, while internodes far away were less affected, implicating a signal gradient emanating from the apical meristem which determines the cell division and elongation potential of internodes underneath. The activation of *DWT1* at early reproductive stage may be required for preventing the production of a mobile growth inhibitor, which prevents the premature elongation of the internodes, or may promote the production of a growth promotion signal. Such alteration may enhance the responsiveness of cells in the upper internodes to GA or cytokinin.

A function in inter-organ coordination has recently been shown for a receptor-like kinase homologous to *Arabidopsis* CLAVATA1, named HYPERNODULATION ABERRANT ROOT FORMATION1 (HAR1). HAR1 is an important regulator of shoot-to-root communication during root nodulation in *Lotus japonicas*. It was proposed that HAR1 negatively regulates nodulation by promoting the production and transportation of a putative shoot-derived inhibitor [54], [55]. As a homolog of the CLAVATA1 downstream component WUS, DWT1 may regulate intra-panicle uniformity also by controlling long-distance coordination between the panicle and internodes or between main shoot and tillers. The nature of the mobile signal, if involved, and the molecular mechanism by which *DWT1* ensures uniform tiller growth in rice require further study. Nevertheless, our study establishes *DWT1* as a key genetic component responsible for uniform tiller growth in domesticated rice. Whether *DWT1* is responsible for this trait during rice domestication and whether it can provide or improve tiller uniformity in other crops are also outstanding questions for future study.

## Materials and Methods

### Plant materials and growth conditions

All plants (*Oryza sativa*) were grown in the paddy field of Shanghai Jiao Tong University. The 11 wild rice strains (Table S6) were ordered from International Rice Research Institute. For GA treatment, wild type and *dwt1* mutants were grown in the paddy field, and at internodes elongation stage, GA_3_ (100 µM) was sprayed to the plant for 3 times at a 2-day interval within 6 days. For the control, 1 mL of 95% alcohol was added to 1L sterilized water. The length of culm and the internode was measured 6 days after treatment, and the final elongation pattern were counted after heading (20 days after treatment). The *Nicotiana benthamiana* (tobacco) plants were grown in the green house under 16 hours light-long day conditions.

### Cell number calculation in the internodes and statistical analysis

Five normal wild-type elongated second internodes and five un-elongated second internodes in the mutant were harvested from different individual plants to count the total longitudinal cell number. The wild type internodes which were separated into 10–15 pieces and the un-elongated *dwt1* internodes at the corresponding stages based on the size of wild-type internodes were embedded into the paraffin and sectioned longitudinally. The cell number of each piece in the longitudinal direction between two vascular bundles were counted under microscope (Motic B3) and calculated. The statistical tests were performed by Student's *t*-test, and the variation is expressed as standard deviation (SD).

### Genotyping of double mutants

The *dwt1* mutant was crossed with the *slr1* mutant. *dwt1*-like and *slr1*-like mutants in F2 generation were genotyped by sequencing. The genotyping fragments for *SLR* (nucleotides 569–1325 of the coding sequence) and *DWT1* (nucleotides 508–1662 of the coding sequence) were amplified by PCR, and sequenced. Three *dwt1slr1* double mutants were identified from 17 *slr1*-like plants in 100 F2 plants. The genotyping primers are listed in Table S7.

### Cloning of *DWT1* and complementation

The F2 mapping population was generated from a cross between *dwt1* mutant (*Oryza sativa ssp. japonica*) and *Longtepu B* (*Oryza sativa ssp. indica*). A 6.7-kb genomic fragment containing the open reading frame, 2.5-kb upstream and 0.6-kb downstream sequences of the *DWT1*, was obtained by PCR from BAC clone OSJNBb0063G05. The genomic fragment was inserted into a binary vector pCAMBIA1301, then was introduced into the *dwt1* mutant by the Agrobacterium-mediated transformation method [56]. The pDWT1: DWT-YFP was constructed by fusing an YFP to the C-terminal of *DWT1* cDNA, which was driven by the native *DWT1* promoter. This fragment was then inserted to the binary vector pCAMBIA1301 and transformed into the wild type. The SNP dataset was obtained from Rice Genome Knowledgebase (RGKbase, http://rgkbase.big.ac.cn/RGKbase/index.php) [57]. SNP sites within a specific region in the genome were extracted using customary Perl script according to gene feature file (GFF) of MSU 6.1 annotation. (ftp://ftp.plantbiology.msu.edu/pub/data/Eukaryotic_Projects/o_sativa/annotation_dbs/pseudomolecules/version_6.1/).

### Phylogenetic analysis

The amino acid sequences were aligned using MUSCLE 3.6 with the default settings [58], and then adjusted manually using GeneDoc (version 2.6.002) software (Pittsburgh Supercomputing Center; http://www.psc.edu/biomed/genedoc/). Using the MEGAsoftware (version 3.1; http://www.megasoftware.net/index.html) and based on full-length protein sequences, midpoint-rooted neighbor-joining trees were constructed with the following parameters: Poisson correction, pairwise deletion, and bootstrap (1000 replicates; random seed) [59]. The accession numbers are list in Table S8.

### Transient transformation

The *35S::DWT1-YFP* vector was transformed into *Agrobacterium tumefaciens* strain GV3101, and the cultured bacteria were infiltrated into young leaves of 4-week-old tobacco plants. YFP fluorescence was visualized with a confocal scanning microscope (Leica TCS SP5 II) 40–48 hours after infiltration [60].

### Total RNA isolation and quantitative RT-PCR analysis

Total RNA was extracted from different tissues of wide type and *dwt1* mutant. The first-strand cDNAs were synthesized using MLV reverse transcriptase (Ferments). Quantitative real-time PCR analyses were performed on a CFX96 (Biorad, US) using a SYBR green detection protocol according to the manufacturer's instructions. The RT-PCR was repeated at least three times for separately harvested samples. The primers used in this article are listed in Table S7.

### Western blot

Total proteins were extracted from internodes, leaves and panicles of wild type with 2× SDS loading buffer, separated on SDS/PAGE gels, transferred to a nitrocellulose membrane, and hybridized with anti-DWT1 antibody. The DWT1 antibody was prepared by Shanghai ImmunoGen Biological Technology and used at 1∶1000 dilution in the immunoblot analysis. Anti-β-tubulin was purchased from Santa Cruz Biotechnology and was used at 1∶1,000 dilution in the experiment.

### Microarray analysis

Total RNA were isolated from elongating internodes (Length = 1.0 cm) of *dwt1* mutant and wild-type plants using TRIzol (Invitrogen). Using the standard Affymetrix protocol, three biological repeats of the microarray experiment were performed with Affymetrix rice genome array by Gene Tech Biotechnology Company, which contains probe sets to detect transcripts from all of the high-quality expressed sequence from the entire rice genome. Significance analysis of microarray was used to identify differentially expressed genes. Genes with at least a threefold change in expression (P values<0.001) were chosen for additional analysis.

### GO enrichment analysis

GO annotations of Microarray genes were downloaded from NCBI (http://www.ncbi.nlm.nih.gov/), UniProt (http://www.uniprot.org/), TIGR (rice.plantbiology.msu.edu/) and the Gene Ontology (http://www.geneontology.org/). The “elim Fisher” algorithm was used to do GO enrichment test [61]. Gene ontology categories with an adjust p-value<0.05 were reported.

### *In situ* hybridization

*DWT1*-specific fragment (nucleotides 1458–1701 of the coding sequence) was amplified by PCR from the cDNA clone and then inserted into pBluescript SK(+) (Stratagene). Preparation of probes and the *in situ* hybridization were performed according to the previous description [62].

## Supporting Information

Figure S1 *dwt1* displays an elliptic transverse shape in the un-elongated internode. A and B. Freehand transverse section of the third internode of wild type (A) and *dwt1* (B) at mature stage. Bar = 500 µm.
(TIF)

Figure S2 Alignment of the amino acid sequences of DWT1 and known WOX9 proteins. Alignment of the amino acid sequences of DWT1 and a series of known WOX9 proteins, STIP, STPL, EVG, SOE, and SLWOX9/COMPOUND INFLORSCENCE, using AtWUS protein as control. Red box square indicates the homeobox domain. Purple and blue squares indicate the conversed motif in the N-terminus and C-terminus, respectively. The accession numbers of the proteins are listed in Table S8.
(TIF)

Figure S3 Phylogenetic analysis of the WOX8/9 subfamily. The phylogenetic tree summarizes the evolutionary relationships among members of WOX8/9 subclass, and the neighbor joining (NJ) bootstrap values are shown in the figure. The AtWUS protein and AtWOX11/12 proteins are used as out-groups. The red branches show the protein from dicot plants, and the blue branches show the proteins from the monocot plants, and the yellow branches show the WOX11/12 subclade. The DWT1 protein is highlighted with a green square. The accession numbers of the proteins are listed in Table S8.
(TIF)

Figure S4 Nucleotide polymorphisms in the *DWT1* coding sequence. The blue box indicates the reference sequence of cultivated rice. The red box indicates the nucleotide polymorphism in wild rice lines.
(TIF)

Figure S5 Expression of *DWT1*, *DWL1* and *DWL2*. mRNA levels of *DWT1*, *DWL1* and *DWL2* in the elongating internode, panicle of wild type, panicle of *dwt1* mutant and the leaf primordium. IN, elongating internode (Length = 5 mm, n = 10); PW, panicle of the wild type in secondary branching stage (n = 50); PD, panicle of *dwt1* mutant in secondary branching stage (n = 50); LP, primordium of the flag leaf (n = 50). *EF1α* gene was used as a control.
(TIF)

Figure S6 Altered expression of genes related to cytokinin homeostasis/signaling in *dwt1*. A. Transcript levels of *OsRR6*, *OsRR9* and *OsRR10* genes in the second internode of the wild type and *dwt1*. B. Transcript levels of O*sCKX4* and *OsCKX9* genes in the second internode of the wild type and *dwt1*. Three sequential developmental stages of elongating internode (Length = 0.5 cm, 1 cm and 3 cm) of wild type and *dwt1* at the corresponding developmental stage of wild type were analyzed. *OsRR9* and *OsRR10* could not be specifically amplified since their high similarity. *EF1α* was used as a control. This experiment was biologically repeated three times, and 5 internodes were used for each biological repeat. Error bars indicate SD. Significant differences from the wild type are marked (* P<0.05, ** P<0.01, Student's *t* test).
(TIF)

Figure S7 The response of wild-type and *dwt1* internodes to GA treatment. A–D: Frequency distribution for the length of the second internodes in the control and GA_3_ treated wild type main shoots (A), wild type tillers (B), *dwt1* main shoots (C) and *dwt1* tillers (D). The wild-type and *dwt1* mutant plants at early reproductive stage were sprayed with GA_3_ or control solution, and 20 internodes were observed for each group, and each data column represents the distribution number of internodes in every 2.5 cm (from 0 cm to 30 cm). E–F: Frequency of internode elongation patterns of both main shoots (E) and tillers (F) under GA treatment. The wild-type and *dwt1* mutant plants at early reproductive stage were sprayed with GA_3_ or control solution, and the internode elongation pattern was observed 20 days after treatment. n = 20. 2^nd^: only 2^nd^ internode short; 2^nd^, 3^rd^: both 2^nd^ and 3^rd^ internodes short; 2^nd^, 3^rd^, 4^th^: all 2^nd^, 3^rd^, 4^th^ internodes short.
(TIF)

Figure S8 Internode elongation in *slr1* and *slr1dwt1* double mutant. A–C. Elongation pattern of elongated internodes in *slr1*mutant (A), *dwt1slr1* mutant (B) and un-elongated internodes in *dwt1slr1* mutant (C). Bar = 1 cm in A and B; 2 mm in C. D–F. Longitudinal sections through the middle of the internode of the *slr1* (D) and elongated internode (E) and un-elongated internode (F). Bar = 100 µm.
(TIF)

Figure S9 Expression of strigolactone related genes is not obviously altered in the *dwt1* seedling. mRNA levels of *HTD1*, *D10*, *D10L*, *D27*, *D14* and *D3* in the seedlings. Leaves, roots and basal shoots (length = 1 cm) were harvested from two-week old wild-type and *dwt1* seedlings grown on the 1/2 MS liquid medium. *EF1α* gene was used as a control. This experiment was biologically repeated three times. Error bars indicate SD. Significant differences from the wild type are marked (* P<0.05, ** P<0.01, Student's *t* test).
(TIF)

Figure S10 No obvious expression change of genes related to cytokinin homeostasis/signaling in the *dwt1* seedling. Expression levels of *OsRR6*, *OsRR9*, *OsRR10*, O*sCKX4* and *OsCKX9* in the wild-type and *dwt1* seedlings. Basal shoots (A, length = 1 cm) and roots (B) were harvested from two-week old of wild-type and *dwt1* seedlings grown on the 1/2 MS liquid medium. *EF1α* gene was used as a control. This experiment was biologically repeated three times Error bars indicate SD. Significant differences from the wild type are marked (* P<0.05, ** P<0.01, Student's *t* test).
(TIF)

Table S1 Identification of the panicle agricultural traits. The primary and secondary branching numbers and spikelet numbers per mature panicle were counted, and the 1000-grain-weigh was measured. The variance between mainstem and tillers of the wild type and *dwt1* were analyzed by the One-way ANOVA test. The different capital letters show the significant differences between the samples. (n = 11 in branching numbers and spikelet numbers counts and n = 3 in grain weight measurement; P<0.01, Student's *t* test).
(XLS)

Table S2 Differential expressed genes (at least threefold change in expression, p-value<0.001) in the shorter internode of *dwt1*.
(XLS)

Table S3 List of genes in the GO categories enriched from the differential expressed genes.
(XLS)

Table S4 Cell elongation related genes in the differentially expressed in *dwt1*.
(XLS)

Table S5 Expression of genes putatively involved in different phytohormones (Gibberellin, Auxin, Brassinosteroids, Cytokinin and Strigolactone) biosynthesis and signaling pathways in shorter internode of *dwt1*.
(XLS)

Table S6 Wild rice strains used for DWT1 sequence analysis.
(XLS)

Table S7 Primers used in this article.
(XLS)

Table S8 Accession numbers used in this article.
(XLS)
