# Supplementary figures and images for: DWARF TILLER1, a WUSCHEL-Related Homeobox Transcription Factor, Is Required for Tiller Growth in Rice
Source: PLoS Genet. 2014 Mar 13;10(3):e1004154. doi: 10.1371/journal.pgen.1004154 (PMC3952828; doi:10.1371/journal.pgen.1004154)

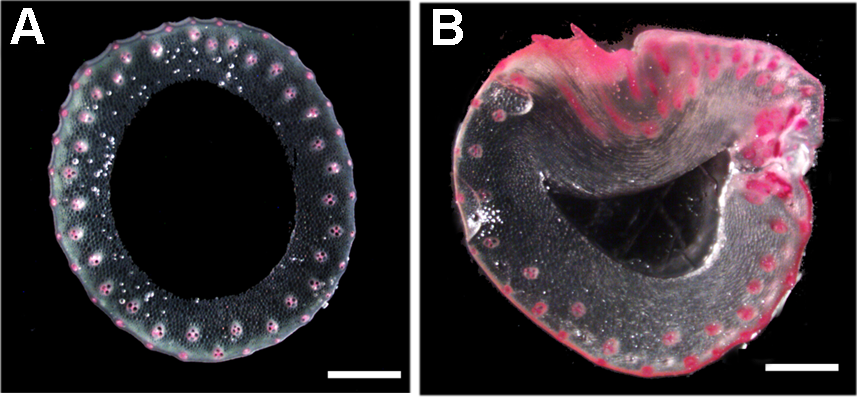

Supplement: Figure S1 — dwt1 displays an elliptic transverse shape in the un-elongated internode. A and B. Freehand transverse section of the third internode of wild type (A) and dwt1 (B) at mature stage. Bar = 500 µm. (TIF) [file pgen.1004154.s001.tif]

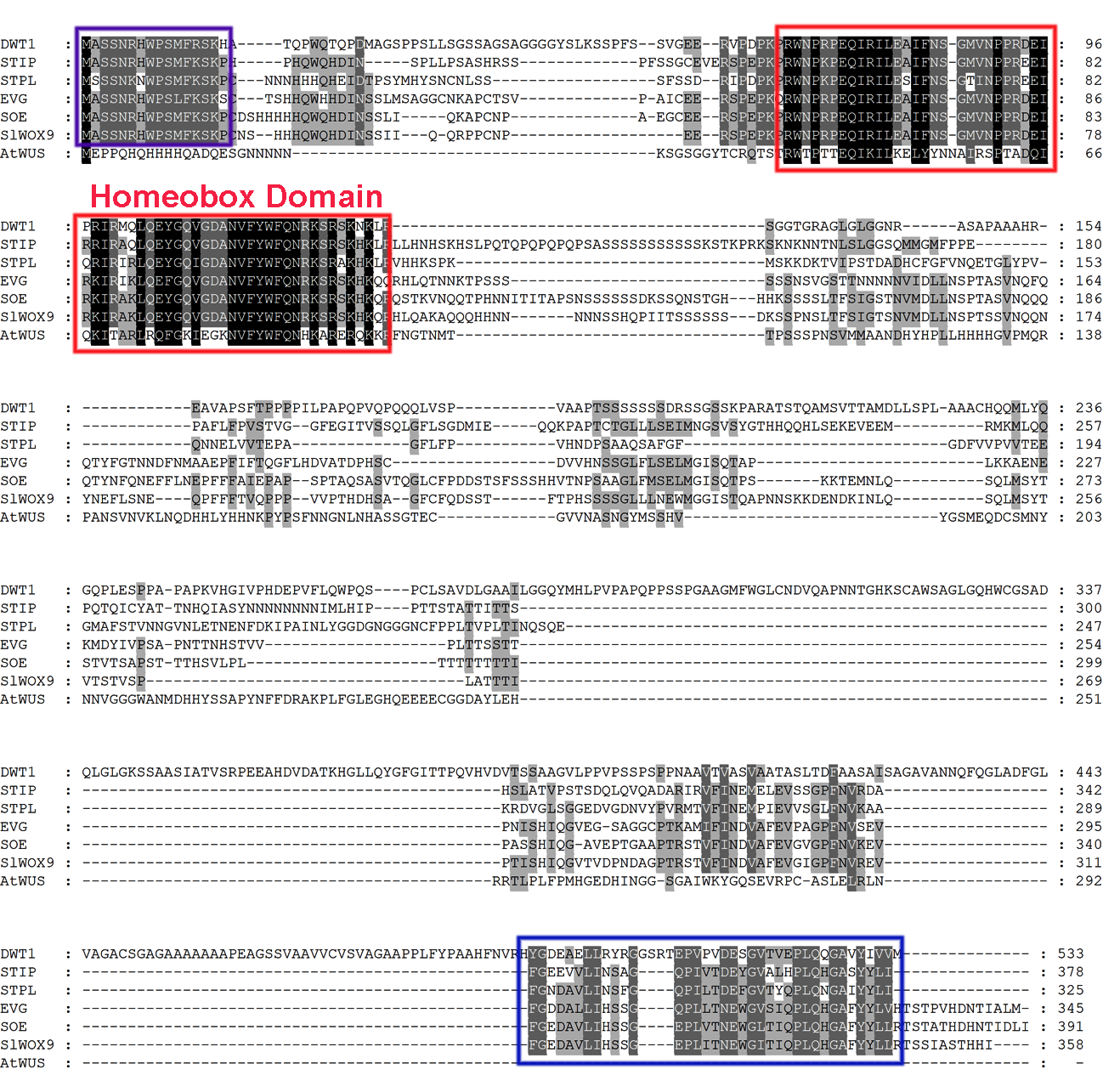

Supplement: Figure S2 — Alignment of the amino acid sequences of DWT1 and known WOX9 proteins. Alignment of the amino acid sequences of DWT1 and a series of known WOX9 proteins, STIP, STPL, EVG, SOE, and SLWOX9/COMPOUND INFLORSCENCE, using AtWUS protein as control. Red box square indicates the homeobox domain. Purple and blue squares indicate the conversed motif in the N-terminus and C-terminus, respectively. The accession numbers of the proteins are listed in Table S8. (TIF) [file pgen.1004154.s002.tif]

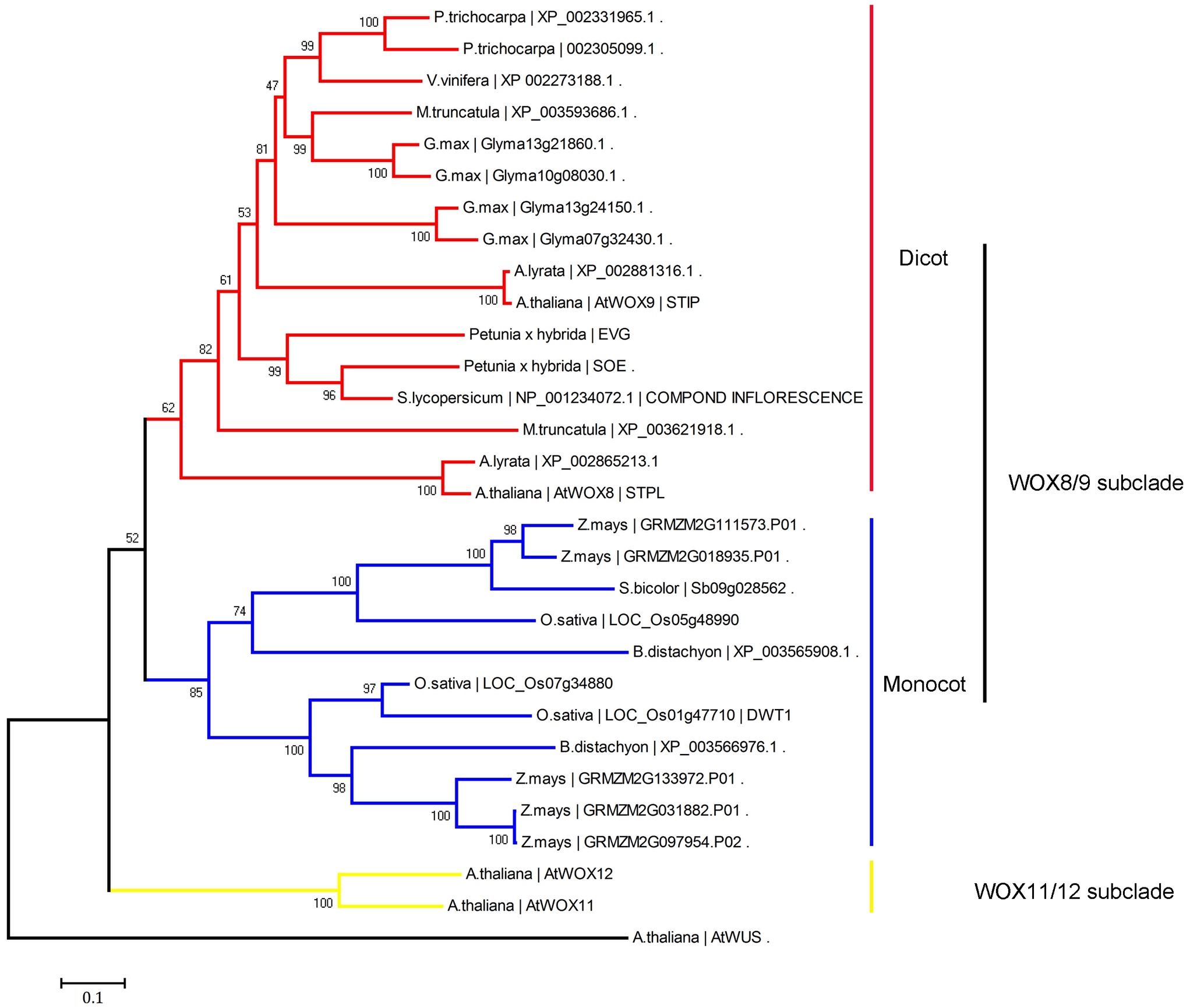

Supplement: Figure S3 — Phylogenetic analysis of the WOX8/9 subfamily. The phylogenetic tree summarizes the evolutionary relationships among members of WOX8/9 subclass, and the neighbor joining (NJ) bootstrap values are shown in the figure. The AtWUS protein and AtWOX11/12 proteins are used as out-groups. The red branches show the protein from dicot plants, and the blue branches show the proteins from the monocot plants, and the yellow branches show the WOX11/12 subclade. The DWT1 protein is highlighted with a green square. The accession numbers of the proteins are listed in Table S8. (TIF) [file pgen.1004154.s003.tif]

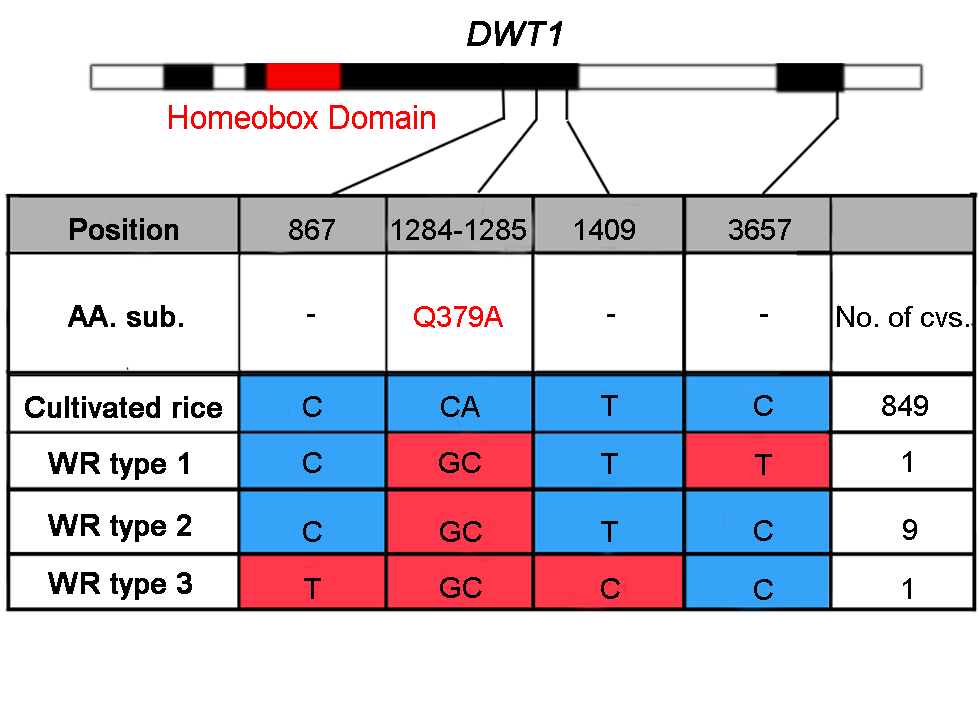

Supplement: Figure S4 — Nucleotide polymorphisms in the DWT1 coding sequence. The blue box indicates the reference sequence of cultivated rice. The red box indicates the nucleotide polymorphism in wild rice lines. (TIF) [file pgen.1004154.s004.tif]

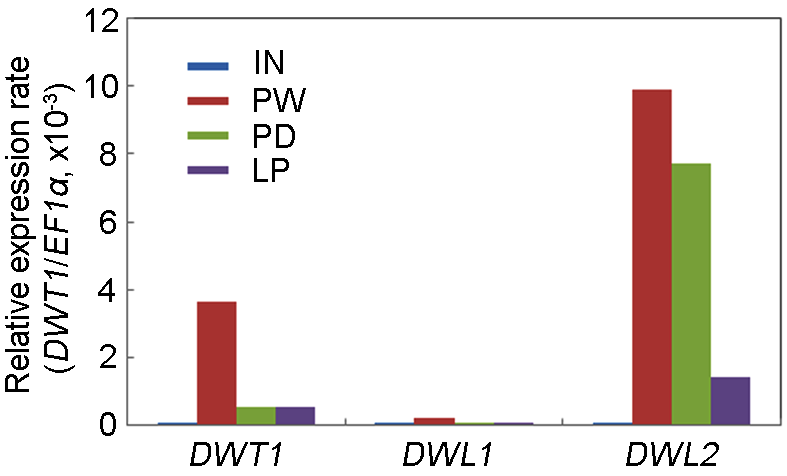

Supplement: Figure S5 — Expression of DWT1, DWL1 and DWL2. mRNA levels of DWT1, DWL1 and DWL2 in the elongating internode, panicle of wild type, panicle of dwt1 mutant and the leaf primordium. IN, elongating internode (Length = 5 mm, n = 10); PW, panicle of the wild type in secondary branching stage (n = 50); PD, panicle of dwt1 mutant in secondary branching stage (n = 50); LP, primordium of the flag leaf (n = 50). EF1α gene was used as a control. (TIF) [file pgen.1004154.s005.tif]

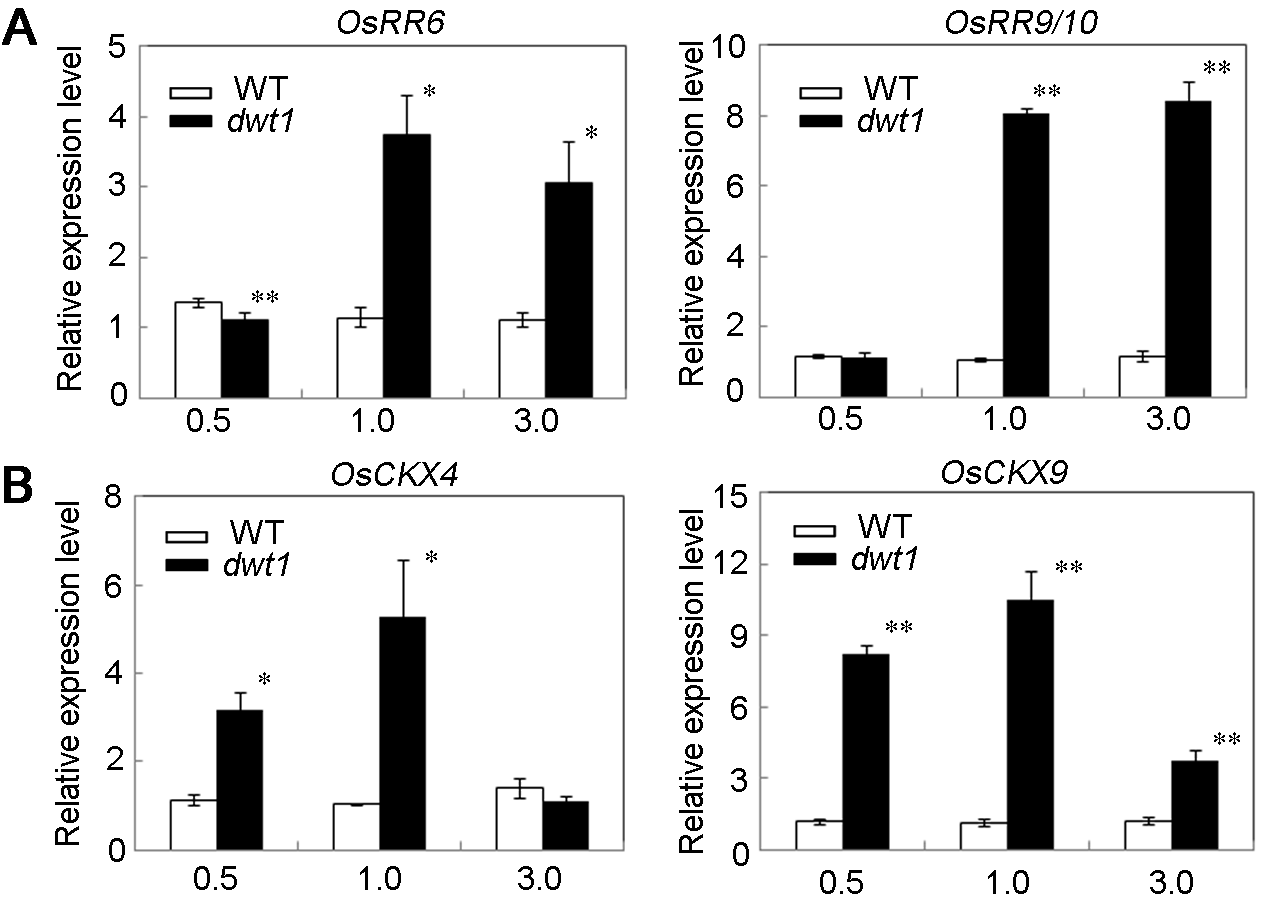

Supplement: Figure S6 — Altered expression of genes related to cytokinin homeostasis/signaling in dwt1. A. Transcript levels of OsRR6, OsRR9 and OsRR10 genes in the second internode of the wild type and dwt1. B. Transcript levels of OsCKX4 and OsCKX9 genes in the second internode of the wild type and dwt1. Three sequential developmental stages of elongating internode (Length = 0.5 cm, 1 cm and 3 cm) of wild type and dwt1 at the corresponding developmental stage of wild type were analyzed. OsRR9 and OsRR10 could not be specifically amplified since their high similarity. EF1α was used as a control. This experiment was biologically repeated three times, and 5 internodes were used for each biological repeat. Error bars indicate SD. Significant differences from the wild type are marked (* P<0.05, ** P<0.01, Student's t test). (TIF) [file pgen.1004154.s006.tif]

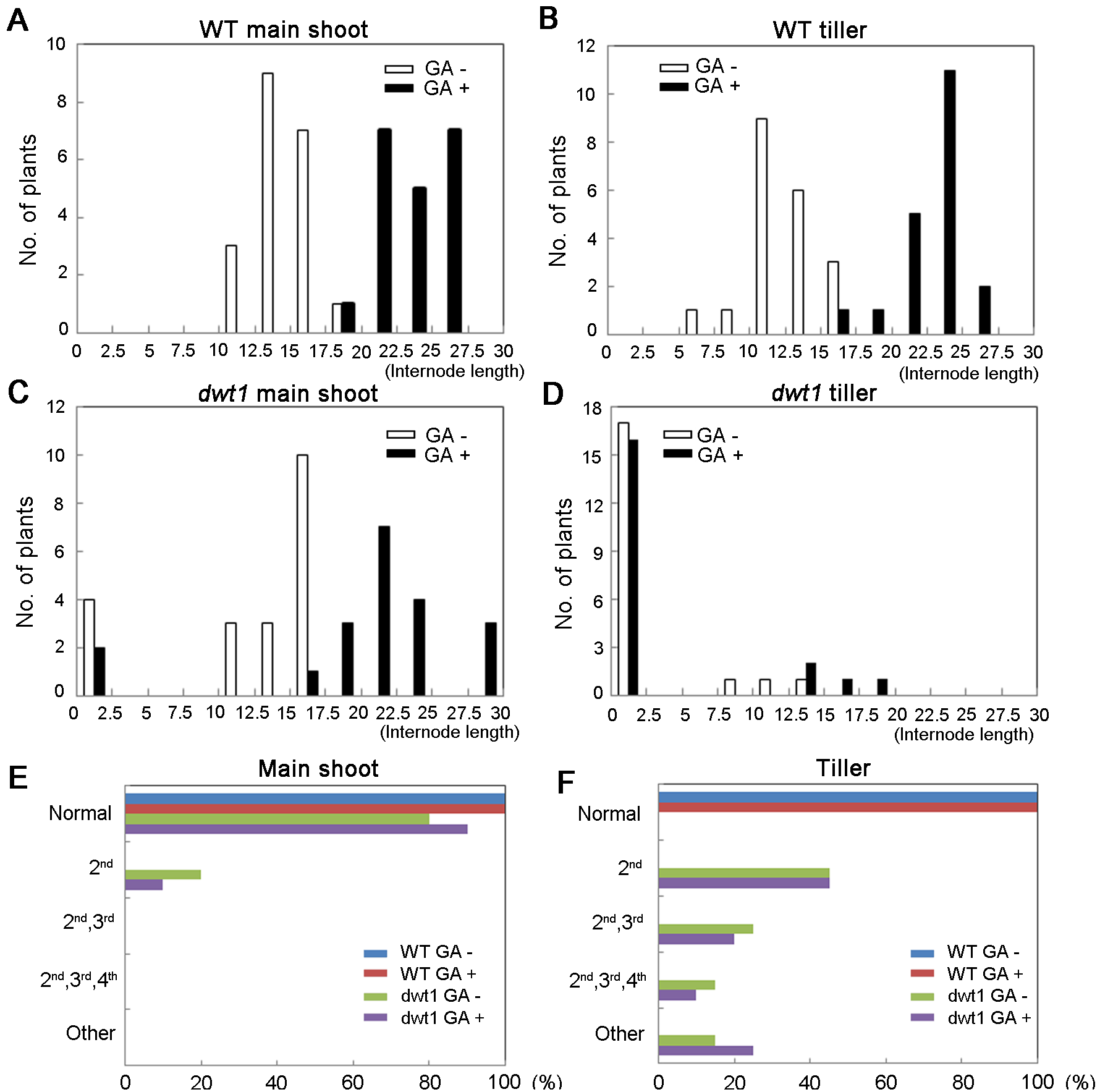

Supplement: Figure S7 — The response of wild-type and dwt1 internodes to GA treatment. A–D: Frequency distribution for the length of the second internodes in the control and GA3 treated wild type main shoots (A), wild type tillers (B), dwt1 main shoots (C) and dwt1 tillers (D). The wild-type and dwt1 mutant plants at early reproductive stage were sprayed with GA3 or control solution, and 20 internodes were observed for each group, and each data column represents the distribution number of internodes in every 2.5 cm (from 0 cm to 30 cm). E–F: Frequency of internode elongation patterns of both main shoots (E) and tillers (F) under GA treatment. The wild-type and dwt1 mutant plants at early reproductive stage were sprayed with GA3 or control solution, and the internode elongation pattern was observed 20 days after treatment. n = 20. 2nd: only 2nd internode short; 2nd, 3rd: both 2nd and 3rd internodes short; 2nd, 3rd, 4th: all 2nd, 3rd, 4th internodes short. (TIF) [file pgen.1004154.s007.tif]

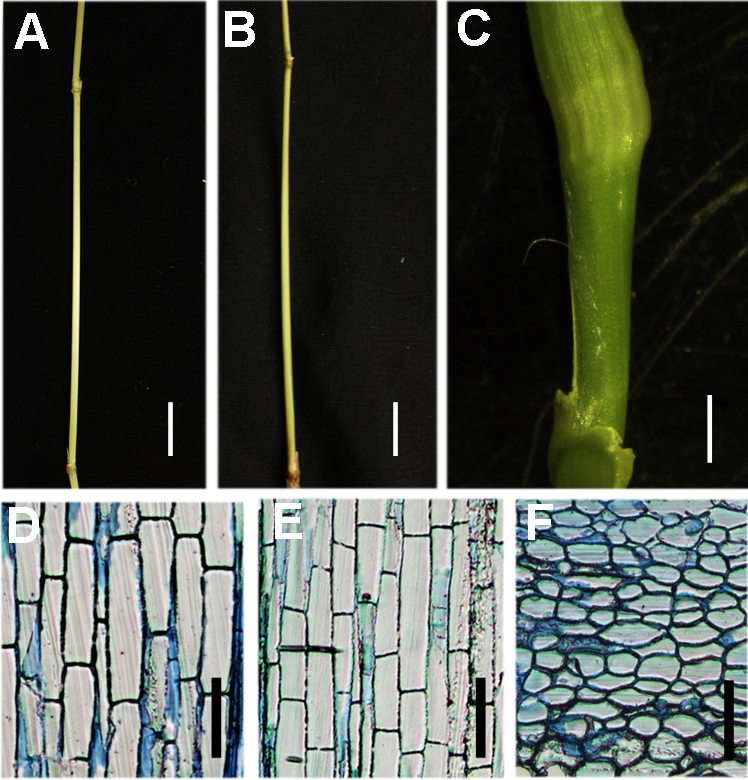

Supplement: Figure S8 — Internode elongation in slr1 and slr1dwt1 double mutant. A–C. Elongation pattern of elongated internodes in slr1mutant (A), dwt1slr1 mutant (B) and un-elongated internodes in dwt1slr1 mutant (C). Bar = 1 cm in A and B; 2 mm in C. D–F. Longitudinal sections through the middle of the internode of the slr1 (D) and elongated internode (E) and un-elongated internode (F). Bar = 100 µm. (TIF) [file pgen.1004154.s008.tif]

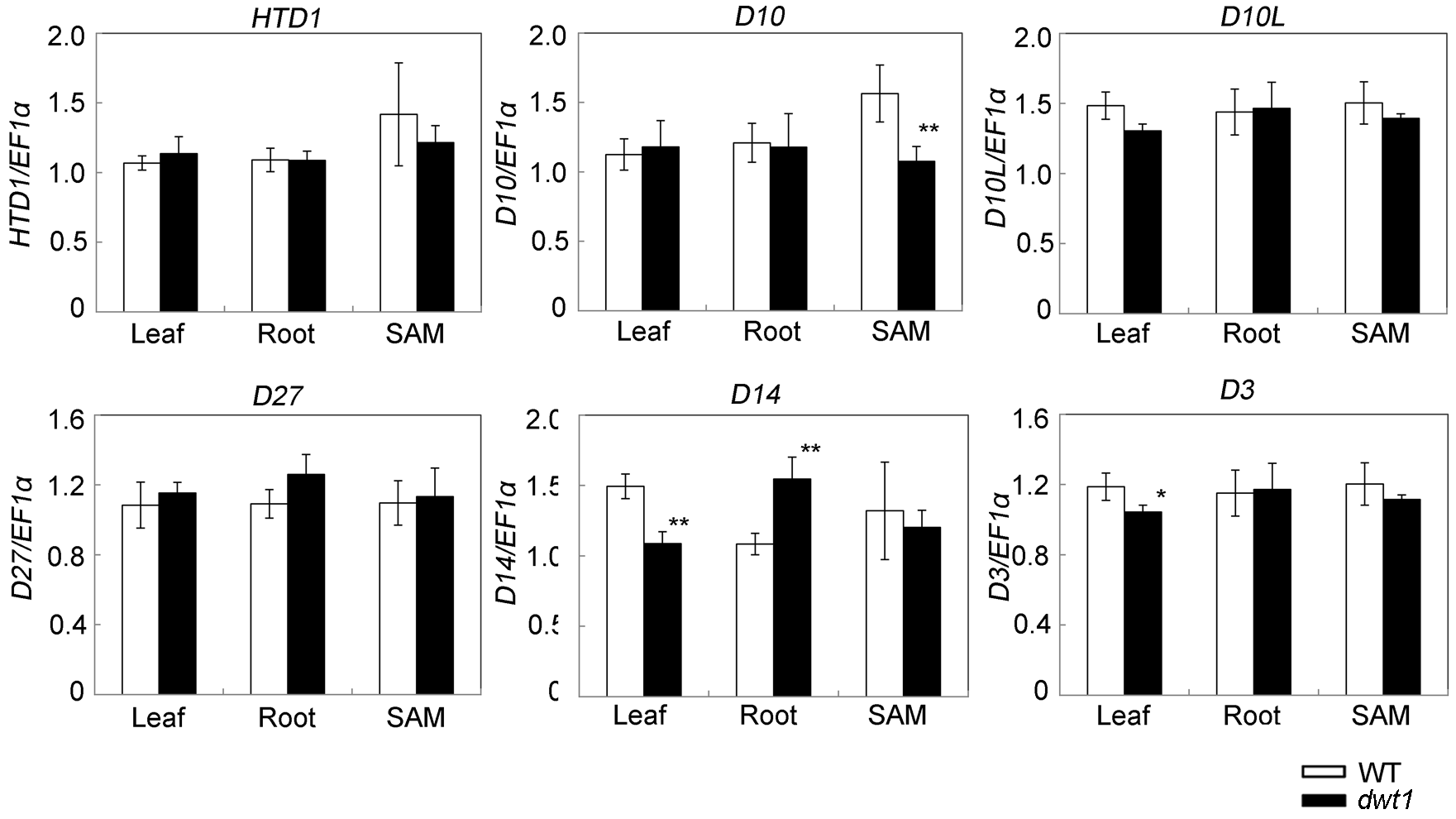

Supplement: Figure S9 — Expression of strigolactone related genes is not obviously altered in the dwt1 seedling. mRNA levels of HTD1, D10, D10L, D27, D14 and D3 in the seedlings. Leaves, roots and basal shoots (length = 1 cm) were harvested from two-week old wild-type and dwt1 seedlings grown on the 1/2 MS liquid medium. EF1α gene was used as a control. This experiment was biologically repeated three times. Error bars indicate SD. Significant differences from the wild type are marked (* P<0.05, ** P<0.01, Student's t test). (TIF) [file pgen.1004154.s009.tif]

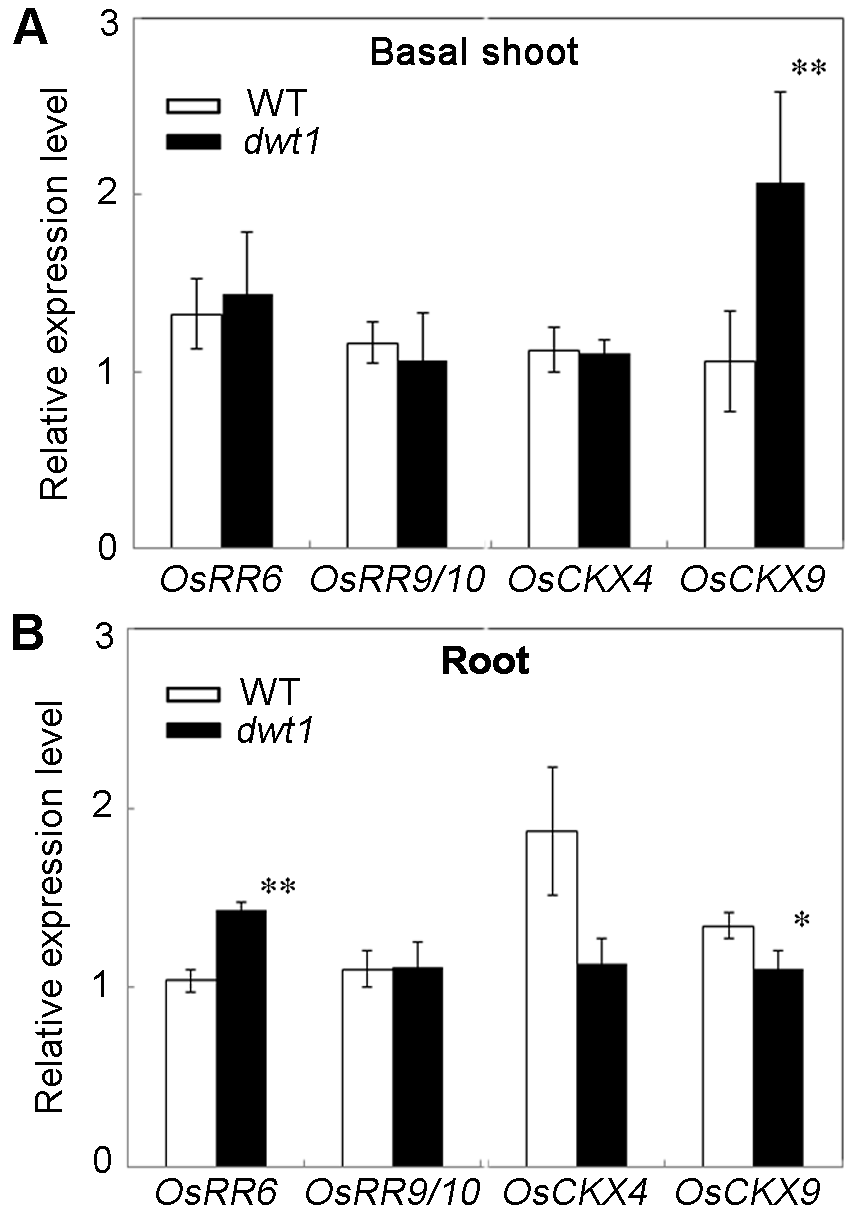

Supplement: Figure S10 — No obvious expression change of genes related to cytokinin homeostasis/signaling in the dwt1 seedling. Expression levels of OsRR6, OsRR9, OsRR10, OsCKX4 and OsCKX9 in the wild-type and dwt1 seedlings. Basal shoots (A, length = 1 cm) and roots (B) were harvested from two-week old of wild-type and dwt1 seedlings grown on the 1/2 MS liquid medium. EF1α gene was used as a control. This experiment was biologically repeated three times Error bars indicate SD. Significant differences from the wild type are marked (* P<0.05, ** P<0.01, Student's t test). (TIF) [file pgen.1004154.s010.tif]
